# Chronic kidney disease mediates cardiac dysfunction associated with increased resident cardiac macrophages

**DOI:** 10.1186/s12882-021-02593-7

**Published:** 2022-01-28

**Authors:** M. A. Mawhin, R. G. Bright, J. D. Fourre, E. I. Vloumidi, J. Tomlinson, A. Sardini, C. D. Pusey, K. J. Woollard

**Affiliations:** 1 Centre for Inflammatory Disease, Department of Immunology and Inflammation, Imperial College London, London, UK; 2 Faculty of Medicine, National Heart & Lung Institute, Imperial College London, London, UK; 3 MRC Laboratory of Molecular Biology, Imperial College London, London, UK; 4 Renal Directorate, Imperial College Healthcare NHS Trust, London, UK; 5 MRC London Institute of Medical Sciences, Imperial College London, London, UK

**Keywords:** uremic cardiomyopathy, macrophages, chemokine, CKD

## Abstract

**Background:**

The leading cause of death in end-stage kidney disease is related to cardiovascular disease. Macrophages are known to be involved in both chronic kidney disease (CKD) and heart failure, however their role in the development of cardiorenal syndrome is less clear. We thus sought to investigate the role of macrophages in uremic cardiac disease.

**Methods:**

We assessed cardiac response in two experimental models of CKD and tested macrophage and chemokine implication in monocytopenic CCR2^−/−^ and anti-CXCL10 treated mice. We quantified CXCL10 in human CKD plasma and tested the response of human iPSC-derived cardiomyocytes and primary cardiac fibroblasts to serum from CKD donors.

**Results:**

We found that reduced kidney function resulted in the expansion of cardiac macrophages, in particular through local proliferation of resident populations. Influx of circulating monocytes contributed to this increase. We identified CXCL10 as a crucial factor for cardiac macrophage expansion in uremic disease. In humans, we found increased plasma CXCL10 concentrations in advanced CKD, and identified the production of CXCL10 in cardiomyocytes and cardiac fibroblasts.

**Conclusions:**

This study provides new insight into the role of the innate immune system in uremic cardiomyopathy.

**Supplementary Information:**

The online version contains supplementary material available at 10.1186/s12882-021-02593-7.

## Introduction

More than 50% of deaths are attributable to cardiovascular diseases (CVD) [1–3]. Even mild chronic kidney disease (CKD) with isolated albuminuria has been associated with a 2- to 4-fold increased risk of cardiovascular events [1]. CVD in renal impairment show manifests in a number of ways, including accelerated atherosclerosis, vascular calcification, abnormal myocardial remodelling, ventricular hypertrophy [4–7]. The mechanistic relationship between renal functional decline and increasing rates of cardiovascular disease and mortality is complex and not fully explained by the frequent co-existence of conventional CVD risk factors such as diabetes, hypertension or dyslipidemia [8]. A number of circulating factors have been implicated as driving CVD in patients with CKD through inflammation, oxidative stress and metabolic derangement such as asymmetric methylarginine or trimethylamine N-oxide [8–10]. Experimental evidence suggests that these factors may be causally related to CVD, but the direction of causality is difficult to determine, as a major confounder is their reduced renal clearance or metabolism with reduced kidney function.

Macrophages have been shown to be important in all stages of inflammatory and fibrotic kidney disease [11]. Their role in cardiovascular comorbidity with kidney disease is less well described, but macrophage activation regulates immune responses during cardiac stress as well as promoting diastolic dysfunction in hypertension and pressure overload models [12–17]. Immune responses are thought to be integral to CKD, considered to be a prototypical example of inflammatory disease [8], and therefore the increased risk of CVD is perhaps unsurprisingly linked to immune cells, as shown for T cells in models of uremic cardiomyopathy [4, 14, 18–20]. However, whether macrophages participate in the development of cardiorenal syndrome is less clear.

Monocytes are heterogeneous leukocytes, with at least 2 functionally distinct populations and 3 different phenotypes in human, termed classical or inflammatory monocytes (‘classical’ and ‘intermediate’ phenotypes) and ‘non-classical’ or patrolling monocytes [21–23]. We and others have identified inflammatory (CD14^++^CD16^+^) monocytes as independent predictors of CVD events in CKD dependently of the lipid profile [23–25]. The relationship between monocyte subset and macrophage phenotype is still to be resolved, however tissue macrophages clearly have diverse functions and are key inflammatory mediators [23]. The heart contains macrophages of mixed phenotypes identified by expression of MHCII and CCR2 [14, 15, 26–28]. They show different ontogeny in the steady state, with evidence of foetal derived self-renewing macrophages and macrophages that are replenished from bone marrow derived monocytes [15–17, 26, 29–32]. Following cardiac injury, monocyte-derived macrophages and resident macrophages have been shown to contribute to the development of cardiomyopathy by promoting inflammation, or limiting inflammation and inducing scar formation [14, 16, 27–29]. The population of macrophages implicated in uremic cardiomyopathy remains to be determined.

In humans, the relationship between CVD and impaired renal function is often confounded by the presence of traditional risk factors such as diabetes, hypertension and smoking which affect both the vascular tree and kidney directly [1, 8]. Animal models provide an opportunity to explore the relationships between CVD and kidney disease in the absence of these confounding risk factors. While mouse 5/6 nephrectomy has been used to model uremic cardiomyopathy [33], the macrophage phenotype and systemic infiltrate has not been fully explored. Furthermore, published data on cardiac phenotypes in non-surgical experimental models of CKD (e.g. folate nephropathy) are sparse. Therefore, we sought to investigate the role of monocytes and macrophages in uremic cardiac disease using both experimental models of CKD.

## Materials and methods

### Animals

Adult mice of 10–16 weeks of age (weighing between 20-28 g) were used for all experiments. Wildtype C57BL/6 mice were obtained from Charles River UK. CCR2 knockout mice, in which homozygous monomeric red fluorescent protein sequence replaces the coding sequence of CCR2, abolishing gene function [34], were purchased from the Jackson Laboratory. All animals were housed in individually ventilated cages. All procedures were carried out according to the Institutional guidelines for the care and use of experimental animals and the ARRIVE guidelines. Animal studies were approved by the UK Home Office. All animals were culled either by exsanguination or intra-cardiac perfusion under non-recovery anesthesia (with isoflurane 3–4% and maintained at 2.5% with an oxygen flow of 1.5 L/min) followed by cervical dislocation.

### Experimental chronic kidney disease models

#### 5/6 nephrectomy (Nx)

**Fig. 1:**
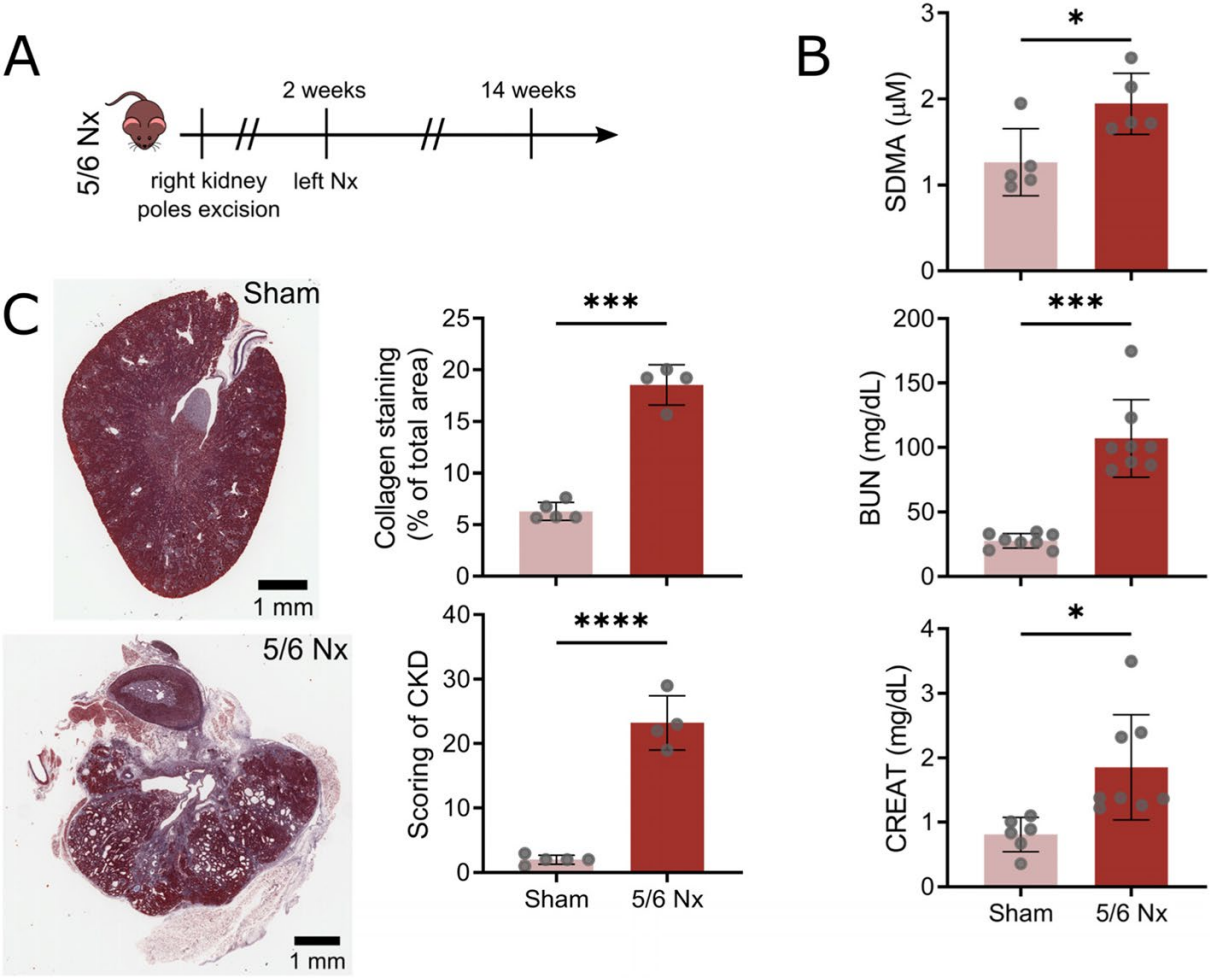
5/6 nephrectomy results in chronic kidney disease. **A** Timeline of the 5/6 Nx procedure showing right kidney excision followed by left Nx at 2 weeks. **B** Plasma SDMA, BUN and creatinine (CREAT) levels in sham and nephrectomised (Nx) mice at 12 weeks. Each point represents individual mice (* *P* < 0.05, *** *P* < 0.001). **C** Representative images of kidney from sham or 5/6 Nx mice (scale bar = 1 mm) stained with Masson’s Trichrome (left). Collagen content as measured by colour deconvolution and CKD score (see materials and methods) (right, *** *P* < 0.001, **** *P* < 0.0001)

Anesthesia was induced with isoflurane 3–4% and maintained at 2.5% with an oxygen flow of 1.5 L/min during the whole procedure. Animals underwent 5/6 Nx or sham surgeries in a two-stage procedure. At stage 1, right kidney poles were removed by excision; at stage 2, after two weeks of recovery, left Nx was performed, as previously described [35] (Fig. 1A). Sub-total Nx mimics the progressive renal failure following loss of renal mass in humans. Using this surgically invasive model, by 8 weeks, glomeruli show mesangial expansion, and focal and segmental glomerular sclerosis involves about 20% of glomeruli, accompanied by early interstitial fibrosis and tubular atrophy. By 12 weeks, reduced renal excretory function and widespread glomerulosclerosis with tubulointerstitial fibrosis are found.

#### Folate induced nephropathy

This is induced by a single intra-peritoneal (IP) dose of folate (240 mg/kg body weight) in vehicle (0.3 mol/L NaHCO3), or vehicle-only in control mice, as previously described [9] (supplemental Fig. 3). Following a single intra-peritoneal injection of high dose folate (240 μg/g), acute tubular cell death and inflammation occurs through luminal crystal deposition and direct tubular toxicity. During the acute phase (2 days to 2 weeks), histological features include gross tubular dilatation, luminal casts and interstitial inflammatory cell infiltrates. In the chronic phase (12 weeks), tubular atrophy and interstitial collagen deposition disrupts the normal cortex architecture and corresponds to loss of kidney function.

For CXCL10 blockade, mice were injected intra-peritoneally with 50 μg of anti-CXCL10 antibody (clone 134,013 R&D systems) or control IgG2a antibody every 4 days for 2 weeks at 10 weeks post-folate injection, as previously described [36].

### Echocardiography

Anesthesia was induced with isoflurane 3–4% and maintained at 2.5% with an oxygen flow of 1.5 L/min during the whole procedure. Echocardiography was performed under anesthesia using a high-resolution VisualSonics Vevo 770 system and a 30 MHz transducer (RMV-707B). See supplemental Material & Methods*.*

### Blood pressure measurement

Anesthesia was induced with isoflurane 3–4% and maintained at 2.5% with an oxygen flow of 1.5 L/min during the whole procedure. The left common carotid artery (LCCA) was isolated from the vagal nerve and surrounding tissues. The LCCA was permanently ligated at the level of the cranial bifurcation and blood pressure was measured by inserting a fiber optic pressure sensor (FISO, Model: FOP-LS-PT9–10) in the LCCA towards the heart. Blood pressure was recorded for a minimum of 5 min and data were analyzed using LabChart6 (AD Instruments).

### Plasma biochemistry

Plasma was obtained by centrifugation from heparinized blood samples and stored at −80 °C. Cytokine and chemokine levels were quantified according to manufacturer’s instruction using LEGENDplex mouse proinflammatory chemokine and virus panel (BioLegend, analytes measured: CCL2, CCL5, CXCL10, CCL11, CCL17, CCL3, CCL4, CXCL9, CCL20, CXCL5, CXCL1, CXCL13, CCL22, IL-1α, IL-6, IL-10,IL-12p70, IFNα, IFNβ, IFNγ, TNFα and GM-CSF). Mouse cardiac troponin-I was quantified by ELISA, according to manufacturer’s instructions (Life Diagnostics). Plasma symmetric dimethylarginine (SDMA) levels were measured using HPLC, blood urea nitrogen (BUN) and creatinine were measured by colorimetric assay (respectively ThermoFisher Scientific and Cayman). 4-hydroxyproline assay was used to assess fibrosis in kidney tissue homogenates according to manufacturer’s instructions (Abcam). Human CXCL10 plasma levels were measured by ELISA, according to manufacturer’s instructions (R&D systems).

### Flow cytometry

Following intracardiac flushing with PBS-5 mM EDTA-20 U/mL heparin, hearts were cut into 1 mm^3^ pieces and digested in RPMI (Gibco) containing collagenase P (10 mg/mL, Roche) and DNase I (1 mg/mL, Roche) for 45 min at 37 °C. Single cell suspensions were filtered on a 40 μm strainer and Fc receptors were blocked using anti-mouse CD16/CD32 antibody (eBioscience). Cells were stained with live/dead stain and with different antibody panels to identify leukocyte subpopulations (supplemental Table 1). Counting beads (BioLegend) were added to quantify normalized cell numbers per mg of tissue. Gating strategy is shown in supplemental Fig. 2. Briefly, single cells were gated on all live CD45^+^ cells as: lineage positive (CD19^+^, CD3^+^, NK1.1^+^); neutrophils (CD11b^+^Ly6G^+^); F4/80^+^MHCII^hi^ or MHCII^lo^ resident macrophages; Ly6C^+^MHCII^hi^ macrophages; CCR2^+^MHCII^hi^ pro-inflammatory macrophages or Ly6C^+^ monocytes as previously described [26]. Blood was treated with red blood cell lysis buffer (BioLegend) and Fc receptors blocked and stained (supplemental Table 1). Blood monocytes and neutrophils were gated as previously described [22]. Briefly, singlet cells were gated on all lineage (CD3, NK1.1, CD19) negative and CD115^+^ monocytes expressing CD11b and Ly6C (see supplemental Fig. 7A). Cells were analyzed on LSR Fortessa. For sorting macrophages from heart tissue, single cell suspensions were sorted using BD FACSAriaII with 85 μm nozzle into Tri-Reagent (Sigma) for RNA isolation, according to manufacturer’s instructions.

For UMAP visualization, data were exported following manual gating with FlowJo and concatenated for 2 representative control and nephrectomised mice using the python library Flowkit. Following pre-processing with hyperbolic arcsine transformation with a cofactor of 150 to obtain a symmetric and linear representation [37, 38] and normalization, parametric UMAP was performed using the python library developed by McInnes [39]. It relies on a neural network of 3 sequential dense layers of 100 neurons activated with rectified linear unit (ReLU) followed by a dense layer of 2 neurons with linear activation. Adam is used for optimization and binary-cross entropy as a loss of function and we employed early stopping to prevent plateauing.

### Histology and immunostaining

Slices (about 2 mm thick) of hearts and other organs were fixed in neutral buffered formalin (10%) and embedded in paraffin. Transverse sections (4 μm) were stained with Masson’s Trichrome, Haematoxylin/Eosin (H/E) and Picrosirius Red (PSR) stain. Whole sections were scanned on Aperio CS2 slide scanner. Collagen staining was quantified on kidney slices stained with Masson’s Trichrome by colour deconvolution, as shown in supplemental Fig. 7B. Kidneys were scored by ranking from 1 to 3 the presence/severity of several features: tubular damages (granular casts, thyroidisation, vacuolation, necrosis, hypertrophy); glomerulosclerosis (focal, global), interstitial infiltrate; interstitial fibrosis; and mineralisation. Cardiomyocyte morphologic parameters were measured on A488-wheat germ agglutinin (WGA) stained whole heart sections [40]. Sections were scanned on Zeiss Axio Observer (Facility for Imaging by Light Microscopy (FILM) at Imperial College) and images tiled using Zen. Using MorphoLibJ watershed segmentation algorithm on Fiji [41], we segmented between 300 and 1000 cardiomyocytes per left ventricle, right ventricle and intraventricular septum for each heart slice and quantified various morphological parameters for each cell. The number of blood vessels was quantified on A647-isolectin B4 (IB4) stained whole heart sections and total collagen on PSR stained whole heart sections scanned under circularly polarised light and tiled on Zeiss Axio Observer.

For cryosections, hearts were fixed in 1% paraformaldehyde, lysine 75 μM, periodate 100 μM in PBS for 4 h at 4 °C, transferred to sucrose 7% overnight, embedded in OCT and snap-frozen in isopentane. Following acetone post-fixation and blocking in PBS-5% BSA-10% NGS, heart slices were stained with antibody against CD68, Ki67 and MHCII (supplemental Table 1) and DAPI in PBS-Triton X-100 0.1%-BSA 5%. After mounting in FluoroShield (Dako), whole slices were scanned and titled on Zeiss Axio Observer. Numbers of macrophages and proliferating macrophages were quantified using an automated macro based on particle analysis on Fiji.

### Quantitative PCR (qPCR)

Hearts were collected and snap frozen. Tissues were disrupted using TissueLyser II (Qiagen) and total RNA was extracted by RNeasy Fibrous Tissue Mini Kit (Qiagen). For sorted macrophages, RNA was collected as above. RNA quality and quantity were assessed by OD reading at 260 nm and 280 nm. RNA was converted to cDNA using SuperScript IV reverse transcriptase (Invitrogen) with Random Hexameres (Invitrogen). Targeted and housekeeping genes were identified using Ensembl database (Mouse (GRCm38.p5) and Human (GRCh38.p13)) and the accession number (RefSeq) used to design qPCR primers using NCBI/Primer-BLAST (supplemental Tables 2 and 3). 1 ng of cDNA was added per reaction and qPCR was performed by triplicates of each sample using 2xSensiMix SYBR Lo-ROX (Bioline) in a AB Viia7-fast block instrument (ThermoFisher). Gene expression was calculated as fold change from control (2^-ΔΔCt^).

### CKD patient recruitment

The study protocol was approved by the Tissue Management Committee of Imperial College Healthcare Tissue Bank according to the Declaration of Helsinki and informed consent was obtained from each donor. The Tissue Bank is authorised by NRES to provide “deemed ethics” by approving applications for use of stored material. CKD patients were recruited from Hammersmith Hospital (mean age 62 ± 15 years). Blood was collected and plasma or serum isolated from patients with CKD stages 3 to 5 according to Kidney Disease Outcomes Quality Initiative guideline classification [42]. Patient characteristics, including monocyte count, statin therapy and cardiovascular risk factors are shown in supplemental Table 4. Sex matched healthy controls were recruited from surrounding labs (n = 24).

### Human cardiac cell culture

Human induced pluripotent stem cell (iPSC)-derived cardiomyocytes, human cardiac microvascular endothelial cells and human cardiac ventricular fibroblasts were prepared and seeded as describes in supplemental Material & Methods*.* Cells were then incubated in their specific media with 10% foetal bovine serum or 10% serum from healthy or CKD donors (n = 2 independent experiments). Serum was collected as above in CKD patient recruitment (sample 38 and 39, in supplemental Table 4). Supernatants were collected and stored at −80 °C until used. RNA was then isolated in cell monolayers, after a thorough rinsing with ice-cold PBS. The RNA isolation was performed using TRIZOL reagent (Invitrogen). Cells were scraped to improve cell lysis and collect as much lysate as possible. Lysates were then stored at −80 °C until further analysis. RT-qPCR was performed as described above and protein secretion in supernatant was quantified using LEGENDplex (IL-4, IL-2, CXCL10, IL-1β, TNF-α, CCL2, IL-17A, IL-6, IL-10, IFN-γ, IL-12p70, TGF-β1, CXCL8).

### Statistics

Following D’Agostino-Pearson omnibus K2 normality testing, comparisons between two independent groups were performed using either two-tailed unpaired t-test, with or without Welch’s correction or Mann-Whitney test. Grouped comparisons were analysed using ANOVA Kruskal-Wallis test and Dunn’s or Dunnett’s post-hoc test for multiple comparison. For PCR, one-sample t test was performed with a hypothetical value of 1. Statistical significance was defined as *P* < 0.05. (*P < 0.05; **P < 0.01, ***P < 0.001). Replicate number (n) for each experiment given in the figure legends.

## Results

### Experimental CKD induced by 5/6 nephrectomy induces cardiomyopathy

Renal impairment by 5/6 Nx (Fig. 1A) resulted in significant increases in plasma markers of CKD at 12 weeks: symmetric dimethylarginine (SDMA), blood urea nitrogen (BUN), and creatinine (Fig. 1B). Consistently, kidneys from 5/6 nephrectomised animals presented high level of interstitial fibrosis, granular casts, tubular dilatation and vacuolation, focal glomerulosclerosis and interstitial infiltrate (Fig. 1C).

**Fig. 2:**
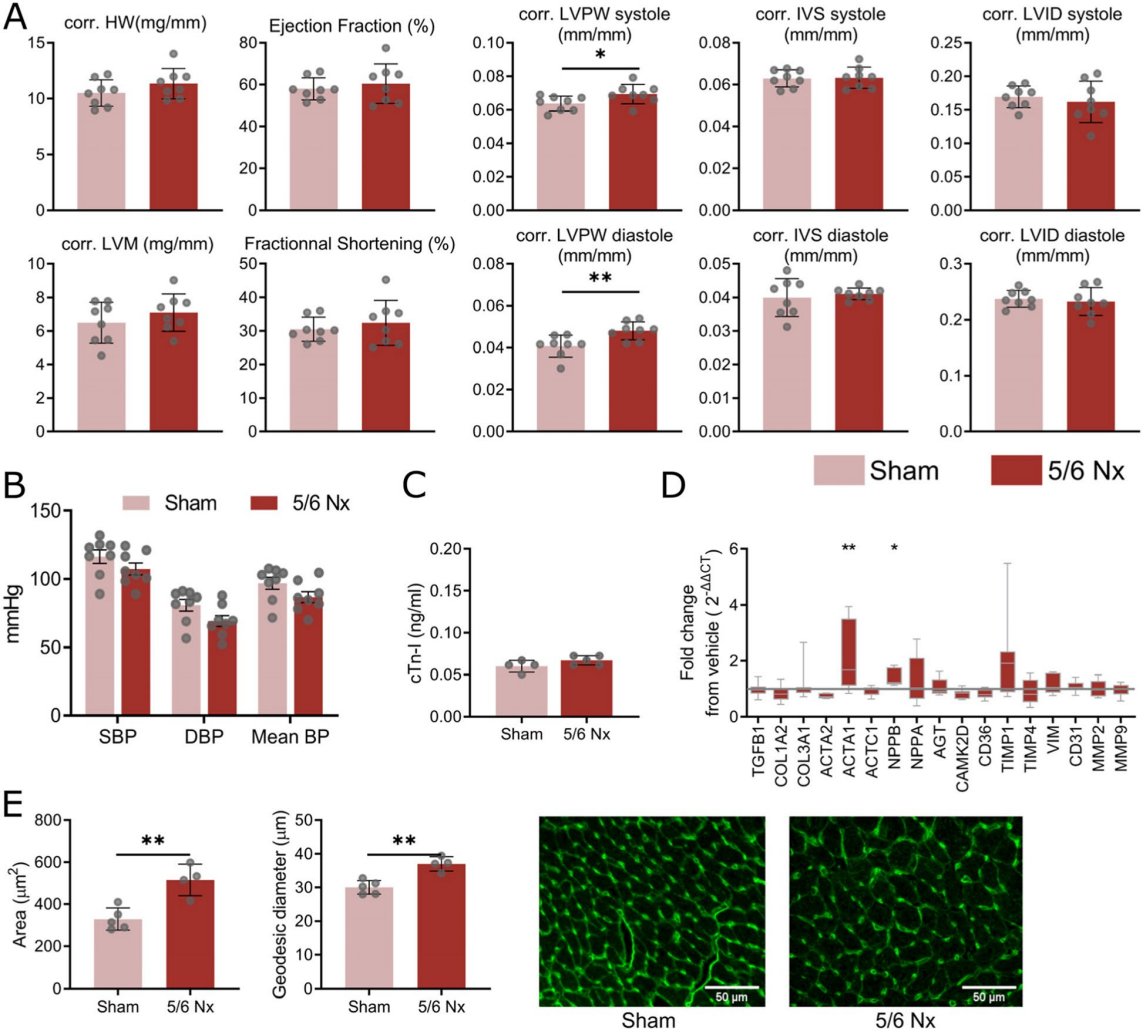
Chronic kidney disease promotes cardiac remodelling. **A** Cardiac remodelling measured by echography in sham and 5/6 nephrectomy (Nx) mice showing heart weight (HW), left ventricular mass (LVM), ejection fraction, fractional shortening, and diastolic/systolic left ventricular posterior wall (LVPW) thickness, intraventricular septum (IVS) thickness, and left ventricular internal diameter (LVID). Parameters are normalised to tibia length. **B** Intracarotid mean, systolic (SBP) and diastolic (DBP) blood pressure expressed in mmHg for sham and 5/6 Nx mice (* *P* < 0.05, ** *P* < 0.01). **C** Plasma levels of cardiac troponin I in ng/mL for sham and 5/6 Nx mice. **D** Fold change from sham in expression of extracellular matrix and cardiac injury genes in hearts of 5/6 nephrectomised mice (12 weeks). Grey line represents no change from sham (* *P* < 0.05, ** *P* < 0.01). **E** Cardiomyocyte cell areas and geodesic diameters (length of the shortest path between two furthest points) in hearts of sham and 5/6 Nx mice and representative A488-WGA staining images (scale bar = 50 μm). Each point represents the mean of at least 300 to 3000 cardiomyocytes per animal (** *P* < 0.01)

To investigate the cardiac phenotype during CKD, we first performed echocardiography on sham and 5/6 nephrectomised mice at 6 weeks and 12 weeks post-Nx. We found an increased left ventricular posterior wall (LVPW) thickness normalised to tibia length both in diastole and systole at 12 weeks between the two groups of mice, while no change in ejection fraction (EF) or fractional shortening (FS) was seen (Fig. 2A). The phenotype did not evolve in both groups from 6 weeks to 12 weeks (supplemental Fig. 1A). These changes are suggestive of cardiomyopathy.

To explore this phenotype further, we assessed whether animals were hypertensive by measuring intracarotid blood pressure. Neither systolic (SBP), diastolic (DBP) or mean blood pressure was increased by Nx (Fig. 2B). This is in agreement with previous reports suggesting that C57/BL6 mouse strain are resistant to hypertension [43, 44]. We also found no increase in plasma cardiac troponin I which might suggest no exacerbation of heart failure [45] (Fig. 2C). We then quantified mRNA cardiac expression of extracellular matrix (ECM) genes and cardiac stress markers and found that both ACTA1 and NPPB expression were elevated in mice with Nx (Fig. 2D), confirming the cardiac remodelling response. There was no clear evidence of endothelial to mesenchymal transition as suggested by the absence of CD31 and Vim overexpression (Fig. 2D). We could not detect changes in number of capillaries or deposition of collagen (supplemental Fig. 1B and C). In agreement with remodelling, we found an increase in cardiomyocyte cell area and geodesic diameter in the whole heart (Fig. 2E) and in the left ventricle (supplemental Fig. 1D and E).

Collectively these results are suggestive of cardiac remodelling resulting in reduced cardiac performance during CKD with 5/6 Nx without hypertension.

### CKD hearts show increased number of proliferating resident macrophage

**Fig. 3:**
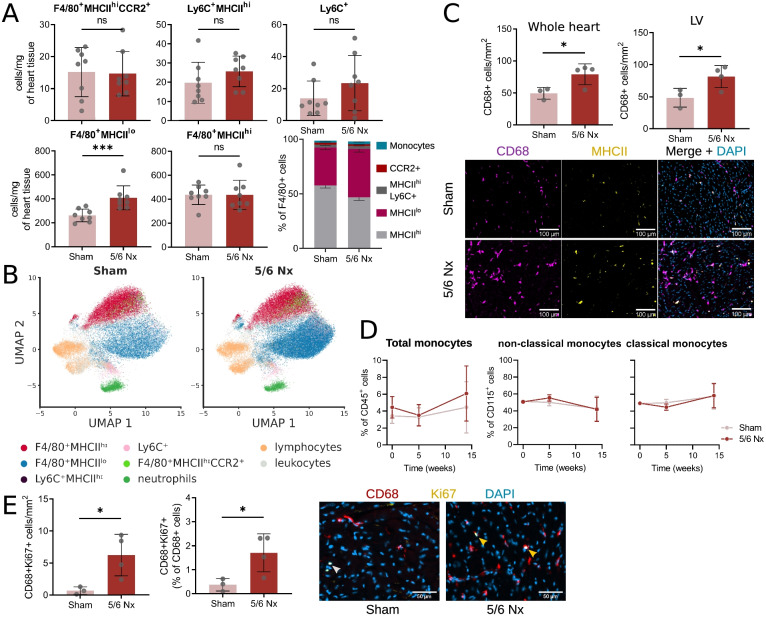
Cardiac macrophage numbers increase during chronic kidney disease. **A** F4/80^+^MHCII^hi^ and F4/80^+^MHCII^lo^ resident macrophage, Ly6C^+^MHCII^hi^ macrophage, F4/80^+^CCR2^+^MHCII^hi^ pro-inflammatory macrophage or Ly6C^+^ monocyte numbers per mg of heart tissue and proportion (stacked barplot) in sham and 5/6 nephrectomised (Nx) mice. (*** *P* < 0.001). **B** Representative parametric UMAP embedding for the visualisation of different leukocyte populations in sham (left) and 5/6 Nx (right) mice. **C** Number of CD68-positive macrophages per mm^2^ of cardiac tissue measured in whole heart slice and in the left ventricle (LV) and representative images showing CD68 MHCII co-staining (scale bar = 100 μm) in sham and 5/6 nephrectomised (Nx) mice (* *P* < 0.05). **D** Total blood monocyte (percentage of all leukocytes) and monocyte subset (non-classical and classical, as percentage of CD115+ monocytes) frequency over 12 weeks in sham and 5/6 Nx mice. Presented as percentage of all leukocytes (total monocytes) or Ly6C^+^ (classical monocytes) or Ly6C^−^ (non-classical monocytes). Mean ± SD at each time point. *n* = 6. E) Proportion of Ki67-positive macrophages in CD68-positive macrophages measured in whole heart slice and representative images showing CD68 Ki67 co-staining (scale bar = 50 μm) in sham and 5/6 Nx mice

Immune cells, in particular macrophages, are known to play a prominent role to cardiac remodelling [4, 13–17, 29]. We thus assessed the immune cardiac phenotype by flow cytometry using the gating strategy shown in supplemental Fig. 2A. Following 5/6 Nx, the number of cardiac macrophages per mg of heart tissue significantly increased (Fig. 3A and B). This increased macrophage population comprises resident F4/80^+^MHCII^lo^ macrophages, known to display predominantly a reparative and regenerative phenotype [28, 46] (Fig. 3A and B). The increase in cardiac macrophage was restricted to the F4/80^+^MHCII^lo^ compartment, as we did not find any increase in F4/80^+^MHCII^hi^ resident macrophages, Ly6C^+^MHCII^hi^ macrophages, F4/80^+^CCR2^+^MHCII^hi^ pro-inflammatory macrophages or Ly6C^+^ monocytes (Fig. 3A and B). Similarly, we did not detect any increased in neutrophils (Ly6G^+^) or lymphocytes (Lin^+^) (supplemental Fig. 2B).

The augmented number of cardiac macrophages was confirmed by immunostaining for CD68 and MHCII of whole heart cryosections (Fig. 3C). We then tested whether this increased macrophage population was associated with a monocytosis. We did not detect any increase in circulating total, non-classical or classical monocytes during the progression of CKD (Fig. 3D). As resident F4/80^+^MHCII^lo^ macrophages have been shown to proliferate locally [15, 26, 31], we next investigated proliferation of CD68 cells by Ki67 immunostaining. We found that the proportion of proliferating cardiac macrophages in nephrectomised mice was increased (Fig. 3E).

To summarise, we found an increase in resident F4/80^+^MHCII^lo^ macrophages in animals during CKD. Proliferation of local resident cells rather than recruitment of blood monocytes might explain the increase in cardiac macrophages during CKD. We next wanted to further investigate the role of monocyte derived cardiac macrophage infiltrate in another CKD model.

### Contribution of macrophage infiltration to cardiac dysfunction in a CKD model of tubular injury

Previous work using other models of cardiac injury such as myocardial infarction [47], pressure overload [14] or hypertension [13], describe a first wave of macrophages being derived from Ly6C^hi^ inflammatory monocytes, which can be abrogated in CCR2^−/−^ mice due to impaired bone marrow egress [48]. We indeed confirmed that CCR2^−/−^ mice were monocytopenic, particularly in classical (Ly6C^hi^) monocytes (supplemental Fig. 4A), as previously described [47].

In order to dissect the role of monocyte derived cardiac macrophage infiltrate during CKD, we choose to employ a consistent non-surgical less severe model of CKD [9, 35, 49]: folate nephropathy using a single intraperitoneal injection of high dose folic acid [9]. As expected, this model resulted in significant but less severe (than 5/6 Nx) plasma increase of SDMA and creatinine, with no perturbation in BUN or plasma cholesterol levels (supplemental Fig. 3A and B). We did note kidney pathology, including increased kidney fibrosis as assessed by hydroxyproline content and tubular atrophy with interstitial collagen deposition (supplemental Fig. 3C). Similar to 5/6 Nx CKD, wild-type mice treated with folate did not present monocytosis (supplemental Fig. 3D).

**Fig. 4:**
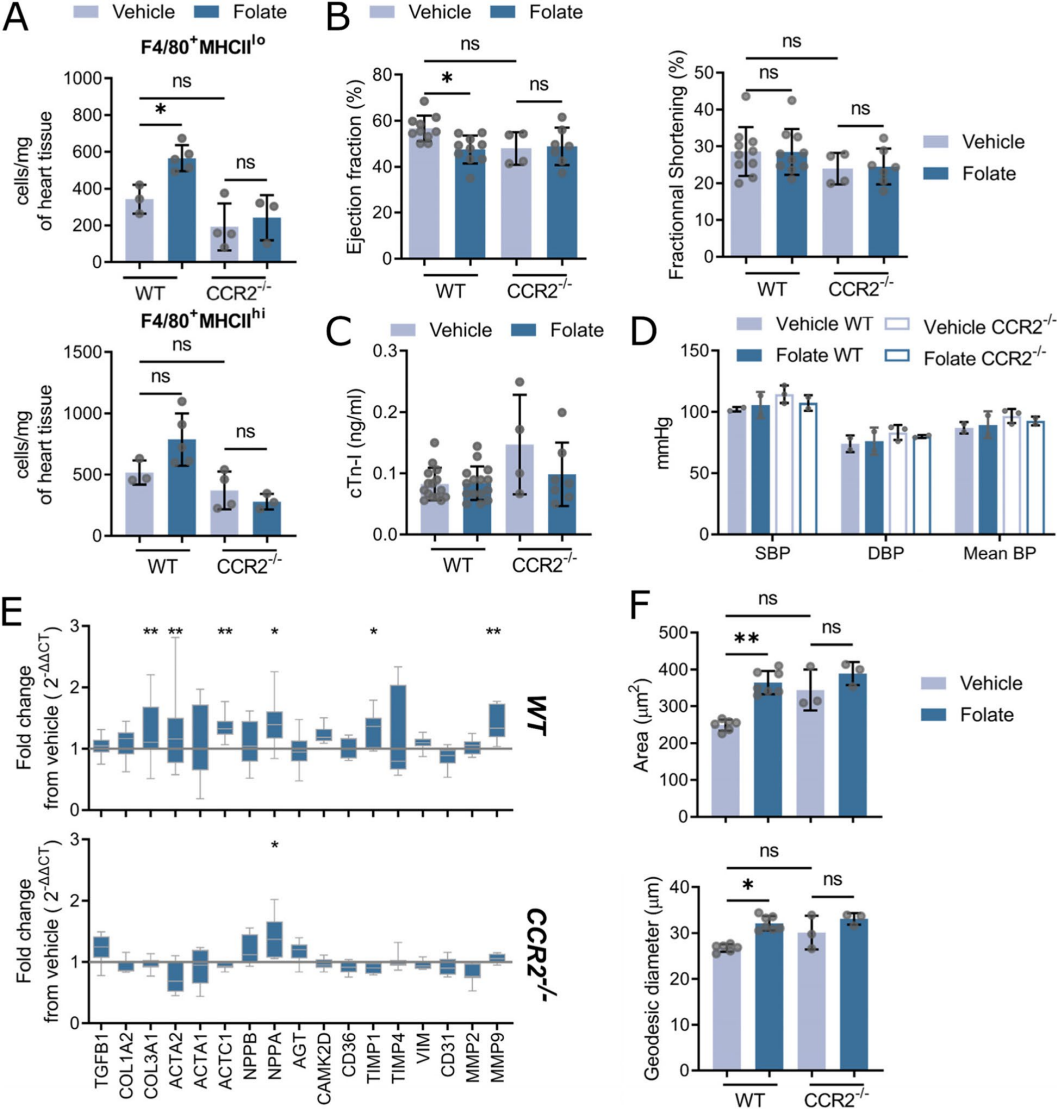
CCR2^−/−^ mice do not show increased numbers of cardiac macrophages and cardiac dysfunction during chronic kidney disease. **A** F4/80^+^MHCII^hi^ and F4/80^+^MHCII^lo^ resident macrophage numbers per mg of heart tissue in vehicle and folate treated (wild-type) WT and CCR2^−/−^ mice (* *P* < 0.05). **B** Ejection fraction (left) and fractional shortening (right) measured by echography in vehicle and folate treated WT and CCR2^−/−^ mice (* *P* < 0.05). **C** Cardiac levels of troponin I in ng/mL in vehicle and folate treated WT and CCR2^−/−^ mice. **D** Intracarotid mean, systolic (SBP) and diastolic (DBP) blood pressure expressed in mmHg in vehicle and folate treated WT and CCR2^−/−^ mice. **E** Fold change from vehicle in expression of extracellular matrix and cardiac injury genes in folate treated WT and CCR2^−/−^ mice. Grey line represents no change from control (* *P* < 0.05, ** *P* < 0.01). **F** Cardiomyocyte cell areas and geodesic diameters (length of the shortest path between two furthest points) in hearts of vehicle and folate treated WT and CCR2^−/−^ mice. Each point represents the mean of at least 1000 to 3000 cardiomyocytes per animal (** *P* < 0.01, *** *P* < 0.001)

We found an increase in cardiac F4/80^+^MHCII^lo^ macrophages in wild-type animal treated with folate which was abrogated in monocytopenic mice (Fig. 4A). Other cardiac population did not show any change following folate treatment in both genotypes (supplemental Fig. 4C). Consistently, wild-type animals exhibited heart failure with reduced ejection fraction (Fig. 4B) with increased expression of ECM and cardiac stress markers (Fig. 4E) along with cardiomyocytes size (Fig. 4F), while no change in cardiac troponin (Fig. 4C), blood pressure (Fig. 4D) or other cardiac parameters was observed (supplemental Fig. 4D). By contrast, CCR2^−/−^ mice were protected from cardiomyopathy (Fig. 4B), showed reduced mRNA expression of ECM genes (Fig. 4E) and cardiomyocyte hypertrophy (Fig. 4F) while still presenting kidney disease (supplemental Fig. 4B). While this suggests a contribution of circulating cells, we still observed macrophages proliferation in the heart of wild-type mice (supplemental Fig. 3E).

In summary, both monocyte infiltration and macrophage proliferation contributed to the development of cardiomyopathy during folate nephropathy.

### CXCL10 plays a role in increasing cardiac macrophages during the development of cardiorenal syndrome

**Fig. 5:**
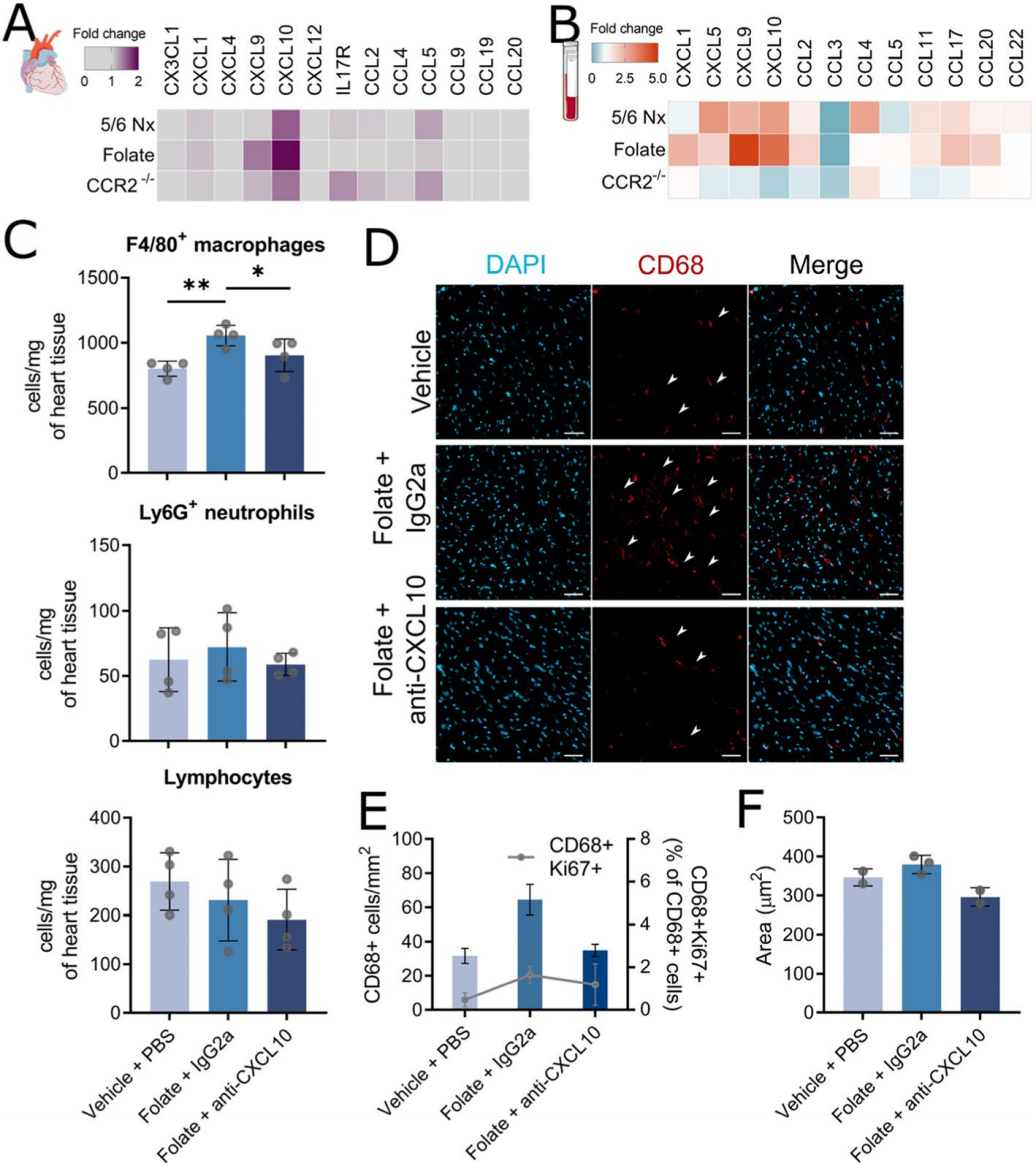
CXCL10 blockade reduce cardiac macrophage numbers but did not protect from cardiac dysfunction. **A** Heatmap showing fold change in cardiac gene expression of chemokines in 5/6 Nx, folate-treated wildtype and folate-treated CCR2^−/−^ mice (to their respective controls). **B** Heatmap showing fold change in plasma chemokine levels in 5/6 Nx, folate-treated wildtype and CCR2^−/−^ mice (to their respective controls). **C** F4/80^+^ macrophage, lymphocyte, and neutrophil numbers per mg of heart tissue in vehicle-treated mice injected with PBS, or folate-treated mice injected with either anti-CXCL10 antibody or control IgG2a antibody(* *P* < 0.05, ** *P* < 0.01). **D** Representative images showing CD68 staining (scale bar = 50 μm) in vehicle-treated mice injected with PBS, or folate-treated mice injected with either anti-CXCL10 antibody or control IgG2a antibody (* *P* < 0.05). E) Number of CD68-positve macrophages per mm^2^ of cardiac tissue and proportion of Ki67-positive macrophages in CD68-positive macrophages (grey line) measured in whole heart slice in vehicle-treated mice injected with PBS, or folate-treated mice injected with either anti-CXCL10 antibody or control IgG2a antibody. F) Cardiomyocyte cell areas in hearts of in vehicle-treated mice injected with PBS, or folate-treated mice injected with either anti-CXCL10 antibody or control IgG2a antibody

To determine potential mechanisms of increased cardiac macrophages during CKD, we quantified cardiac expression and circulating levels of several chemokines and cytokines known to be involved in immune cell trafficking and inflammatory responses. The RNA quantification showed that in both CKD models, the most prominent tissue expressed cardiac chemokine was *CXCL10,* a potential macrophage chemokine [50] (Fig. 5A). To determine if the source was resident cardiac macrophages, a pool of Lin^neg^CD11b^+^F4/80^+^ cells was then sorted from either vehicle control or folate-treated mice. However, these macrophages did not overexpress chemokines (supplemental Fig. 5A). To determine circulating/systemic profile, the plasma levels of chemokines and inflammatory mediators revealed that in both models CXCL9 and CXCL10 were elevated (Fig. 5B). Interestingly, we observed an elevated production in IFN-γ in both CKD models while no major elevation of other cytokines was found (supplementary Fig. 5B). Moreover, in CCR2^−/−^ mice, the levels of plasmatic CXCL10 were decrease linking cardiac macrophage infiltration and proliferation to systemic CXCL10 production.

To further assess the role of CXCL10 in cardiac responses during CKD, we repeatedly injected folate-treated mice with an antibody targeting CXCL10. Mice treated with anti-CXCL10 antibody still presented kidney fibrosis and scored high for CKD (supplemental Fig. 5C). However, treatment with anti-CXCL10 antibody prevented the increase in cardiac F4/80^+^ macrophages as compared to treatment with IgG2a control, while no difference in neutrophils and lymphocytes numbers was found (Fig. 5C). This difference was confirmed by immunostaining (Fig. 5D). However, we could not find a major decrease in proliferating macrophages (Fig. 5E). Anti-CXCL10 limited the cardiac phenotype during CKD, as shown by the non-significant trend to reduce cardiomyocyte cell area (Fig. 5F).

Thus, CXCL10 appears to be involved in the recruitment of macrophages to the myocardium and cardiac phenotypical response during kidney disease.

### A potential role for CXCL10 in human CKD

Our data showed that CKD induced a chemokine axis of overexpression in plasma and cardiac tissue involving CXCL10. As CXCL10 showed the largest increase in cardiac tissue expression (Fig. 5A and B) during CKD, we selected this chemokine to analyse further.

**Fig. 6:**
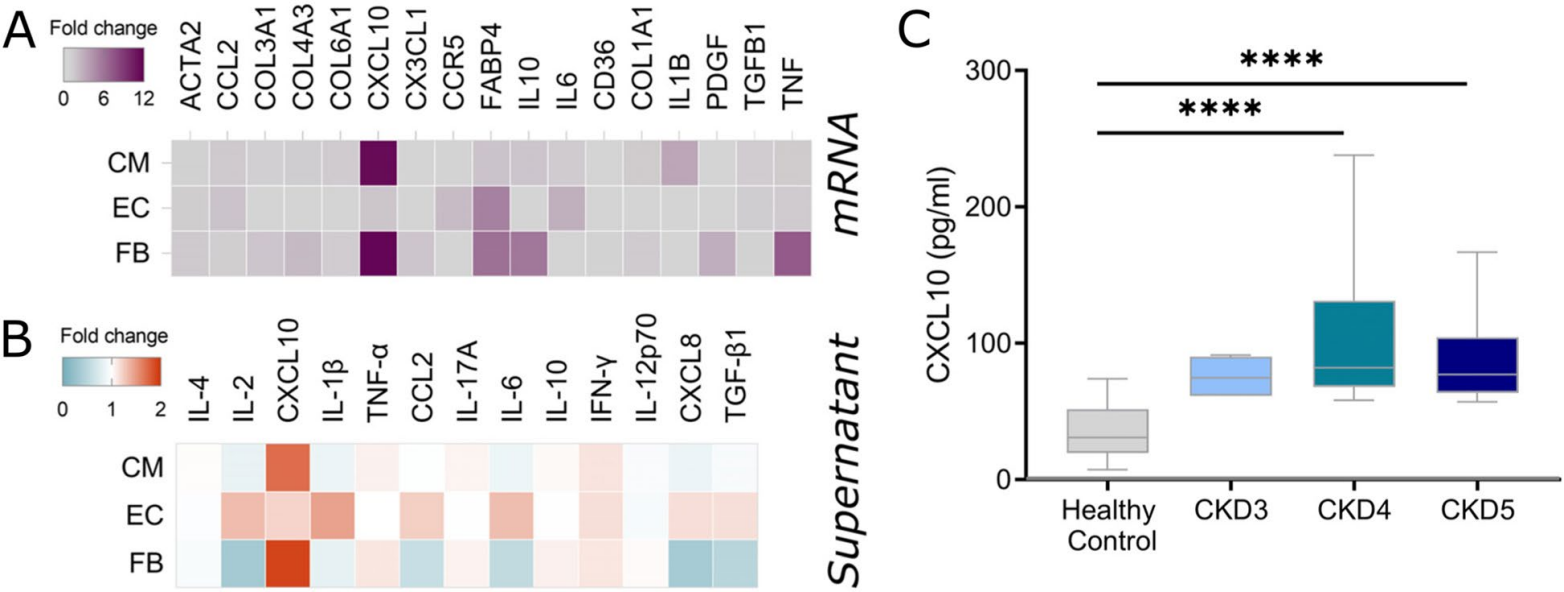
Increase in plasma and cardiac cell expression and production of CXCL10 in human chronic kidney disease. **A** Heatmap showing mRNA expression fold change from healthy control in iPSC-derived cardiomyocytes (CM), human cardiac microvascular endothelial cells (EC) and primary ventricular fibroblasts (FB) treated with serum from healthy or CKD donors. Mean of two independent donors. **B** Heatmap showing protein secretion fold change from healthy control in supernatant of iPSC-CM, EC and FB treated with serum from CKD patients. Mean of two independent donors. **C** CKD patients or matched healthy controls were recruited and plasma levels of CXCL10 measured by ELISA. CKD patients were split into CKD stage 3, 4 or 5. Each individual donor was tested in triplicate. **** represents *P* < 0.0001 from healthy controls

*In-vivo*, we could not detect change in CXCL10 expression in cardiac macrophages (supplemental Fig. 5A). To better understand which cardiac cells could produce CXCL10, we decided to explore the cardiac response *in-vitro* in a proof-of-concept experiment using two CKD donors (see methods). For this, healthy or CKD sera were incubated with 3 different cardiac cell populations: cardiomyocytes (iPSC-CMs); primary ventricular fibroblasts; and microvascular endothelial cells. Incubation of the 3 cell types with healthy serum had marginal effects on both mRNA and protein expression, as seen by comparing control wells with FBS to healthy serum incubation (supplementary Fig. 6A and B). Endothelial cells showed a minimal response to CKD sera with some elevation in IL-1β and IL-6 (Fig. 6A). Interestingly however, we found elevated expression of CXCL10 in both cardiomyocytes and ventricular fibroblasts following treatment with CKD serum as compared to healthy control serum treated cells (Fig. 6A). We also found increased CXCL10 protein production in the supernatant of both cardiomyocytes and ventricular fibroblasts in response to with CKD serum (Fig. 6B).

As a further step towards translating our findings into human disease, we examined plasma expression of CXCL10 in patients (*n* = 37) with CKD stages 3–5, (based on the Kidney Disease Outcomes Quality Initiative guidelines) and compared to healthy controls (*n* = 24). Donor characteristics are shown in supplemental Table 4 including clinical presentation, eGFR, cardiovascular risk factors, blood counts, lipid levels and statin therapy. All CKD patients showed significantly elevated levels of CXCL10 (Fig. 6C) with a mean level of 95.59 ± 41.7 compared to 34.73 ± 19.92 pg/ml in healthy controls. A large difference was particularly seen between CKD stages 4–5 and healthy controls. There were no significant differences in levels of CXCL10 between CKD stages. While the sample size is small, there was no correlation of CXCL10 levels with eGFR and no obvious association of CXCL10 levels with previous cardiovascular events, other cardiovascular risk factors or monocyte counts (data not shown).

Overall, these data suggest overexpression of CXCL10 by cardiomyocytes and cardiac fibroblasts is associated with CKD and possibly uremic cardiomyopathy. This warrants further investigation of CXCL10 as a biomarker and mediator of the cardiovascular risk seen in human CKD.

## Discussion

Despite the well described association between cardiac and renal disease, precise mechanisms underlying this relationship poorly understood [8, 51]. As CKD is associated with systemic inflammation [52] and heart dysfunction with immune responses [28, 30], we wanted to explore the role of cardiac macrophages in the cardiorenal syndrome. We have found in situ proliferation of cardiac resident macrophages in two models of CKD along with bone-marrow derived replenishment of cardiac macrophages in folic acid nephropathy. Monocytopenia prevented the increase in resident macrophages and changes in cardiac microenvironment during nephropathy. This increase in resident cardiac macrophages following renal impairment was dependent upon CXCL10 signalling, but independent of hypertension or renal dysfunction. Consistently, CXCL10 was found to be increased in human CKD plasma and its production was stimulated by CKD serum in human cardiomyocytes and fibroblasts.

Various immune cells have been involved in the progression of heart diseases [4, 12, 14, 18, 20, 28], in particular macrophages and T cells. In our setting, we only observed an increase in MHC^lo^ macrophages and did not observe changes in other cardiac immune cells. A recent study employing young 129x1/SvJ mice found that in early kidney disease, T cells are predominant in the hearts of uremic mice [4]. In transaortic constriction (TAC) models, T cells have been shown to be crucial for macrophage increase and cardiac dysfunction to occur [18, 20]. T cells have also been shown to be activated by macrophages, as antigen presenting cells, resulting in transition from hypertrophy to heart failure [14, 18]. Therefore, these studies may suggest a feed forward loop where macrophages promote T cell expansion which further promotes macrophage infiltration. As our study is in late-stage disease in older and more resistant C57BL/6 animals, this could explain the absence of T cell involvement in our models, in a more hypertrophic and compensated phenotype. Similarly, the absence of hypertension in our models could explain the absence of T cells as they are involved in hypertensive responses [4, 14]. The resistance of nephrectomised C57BL/6 to hypertension is still controversial [43, 44, 53], therefore, it would be interesting to explore at the potential involvement of T cells in hypertensive prone strains, following angiotensin II treatment or at different timepoints.

In most models of severe cardiac dysfunction, such as myocardial infarction (MI) or TAC, CCR2-derived macrophages tend to be the predominant cells in the heart following injury [14, 47]. Consistently, in two models of diastolic dysfunction, i.e. hypertension induced by salty drinking water with unilateral nephrectomy and chronic exposure to aldosterone (SAUNA) and physiological aging, cardiac macrophage expansion has been shown to rely on CCR2 [13]. In our setting, CCR2^−/−^ animals showed reduced F4/80^+^MHC^lo^ cardiac macrophage content and were protected from cardiomyopathy. However, in wild type animals we could not detect any increase in cardiac F4/80^+^CCR2^hi^ macrophages or Ly6C^hi^ monocytes at 6 weeks (supplemental Fig. 3) and 12 weeks post-nephrectomy or folate. Similarly, we did not observe monocytosis or increased plasma and cardiac expression of CCL2 in both models. The former could be explained by the lack of hypercholesterolemia in these animals, as previous clinical studies have shown that monocytosis is linked to elevated cholesterol in CKD patients [24]. Therefore, F4/80^+^CCR2^+^ macrophages might infiltrate the heart at earlier stages in our models and contribute to the pool of resident cardiac macrophages by down regulating CCR2 expression, as has been shown for F4/80^+^MHC^hi^ cardiac macrophages in MI and hypertensive models [15, 17, 26]. Interestingly, cardiomyocyte hypertrophy but not fibrosis has been shown to be independent of F4/80^+^CCR2^+^ influx in TAC models [14, 16]. This supports the hypertrophic phenotype we observed where uremic hearts presented cardiomyocyte hypertrophy and increased left ventricular thickness. Consistently, F4/80^+^MHC^lo^ macrophages are found to be more reparative, with functions involving phagocytosis of dying cells and local homeostasis [28, 46]. These macrophages have also been shown to counteract fibrosis in hypertensive models while macrophage IL10 (which we found elevated in the folate model) has been shown to lead to impaired myocardial relaxation [13]. Interestingly, in our hands CCR2 deficient mice still exhibited significant renal disease and therefore CCR2 deficiency does not appear to improve renal function in our model of CKD. However, pharmacological inhibition of CCR2 has shown renal protection in experimental and clinical diabetic kidney disease [54, 55]. Therefore, our data may highlight differences in the role of CCR2 in different causes of chronic kidney injury.

We show that CKD mediates increased cardiomyocyte size and diameter along with extracellular matrix gene expression. While we did not see an obvious increase in cardiac fibrosis, nephrectomised mice showed increased in ventricular thickness indicating significant cardiac remodelling, which is consistent with other CKD models and CKD patients [4, 52]. In the folate nephropathy model, however, the cardiac dysfunction showed a reduction in ejection fraction, to levels seen in other mouse CKD models [56], suggesting a transition to systolic dysfunction as seen in TAC^14^. Interestingly, we show 5/6Nx had less severe cardiac dysfunction, and while this model is associated with acute reduction of kidney function, folate mediates acute tubular injury [35]. Therefore, acute tubular damage induced by folate injury in the first weeks, may be mediating cardiac injury. In agreement, we found an increase in MMP expression, previously associated with the transition from diastolic to systolic dysfunction [57, 58]. It may be interesting, in future work, to extend our CKD models past 12 weeks to examine if cardiac fibrosis becomes evident.

We have observed a proliferation of cardiac macrophages in two models of uremic cardiomyopathy. The dual contribution of in situ proliferation and monocyte infiltration in the pool of cardiac macrophages has been shown before in hypertensive models [26]. However, the signal driving the proliferation of cardiac macrophages remains unclear. Kidney-derived CSF2/GMCSF has been shown to simulate KLF4-dependent resident macrophage proliferation in the heart following TAC [12, 16]. We did not find any increase in GM-CSF in the plasma of folate-treated or nephrectomised mice. Moreover, of the chemokines that were upregulated during CKD, only CXCL10 was consistently elevated in cardiac tissue and plasma during folate induced nephropathy and after 5/6 nephrectomy. Furthermore, CXCL10 reduced when macrophage infiltrate was inhibited in monocytopenic mice and its blockade prevented cardiac macrophage expansion. The limited effects of CXCL10 blockade on the cardiac phenotype in our model might be due to the small number of animals and the short treatment timespan in our study, which we adapted from previous work [36, 59]. A extended time of treatment could potentially lead to more pronounced effect and warrants further investigation. In pressure overload, CXCL10 has recently been suggested to promote CD4 T cell recruitment [18, 19]. We did not observed an increased number of T cells which suggests that CXCL10 might act by activating local T cells to promote macrophage proliferation or act directly on macrophages [26]. CXCL10 has been shown to be produced by fibroblasts and cardiac macrophages in murine TAC models [19]. It is possible that in our setting, cardiac macrophages stimulate the production of CXCL10 resulting in a positive feedback loop. Interestingly, cardiac tissue macrophages were not responsible for CXCL10 overexpression in folate-treated mice. It would be interesting to explore this hypothesis further in nephrectomised or other CKD models, especially since we found that CXCL10 promoted macrophage expansion.

We investigated whether CXCL10 was also involved in systemic and cardiac responses during human CKD. We found that plasma CXCL10 was increased during CKD and that this increase was more pronounced with more severe disease. To gain insight on how plasma factors in CKD could trigger a response in cardiac cells, we assessed the response of cardiac fibroblasts, cardiomyocytes, and cardiac endothelial cells. We found that these cells responded by expressing pro-fibrotic and pro-inflammatory genes, such as TGFβ1, IL1β, IL10, and IL6. Moreover, we identified that cardiomyocytes were the main cells producing CXCL10 in response to CKD serum. This is consistent with previous studies showing that murine and human cardiomyocytes and fibroblasts produce CXCL10 in response to IFN-γ [19, 60, 61], that we have found in the plasma of uremic mice and the supernatant of serum-treated human cardiac cells in our models. Interestingly, IFN-γ has been shown to be elevated in CKD patients [62]. Further work is now needed to validate elevated IFN-γ as a key trigger in cardiac dysfunction in CKD. Other factors such as mechanical stress or plasma mediators, such as galectin-3 [63] or FGF-23 [64], could also contribute to the cardiac response and further investigation is needed to elucidate their roles.

## Conclusions

Overall, we have shown, for the first time, the dual contribution of local proliferation and monocyte infiltration increased cardiac macrophages in uremic cardiomyopathy. This was associated with cardiac hypertrophy and dysfunction dependent upon CXCL10 signalling. These data provide a first step to comprehending of the complex function of the innate immune system in uremic cardiomyopathy.

## Supplementary Information

**Additional file 1.**

**Additional file 2.**

## Data Availability

All data generated or analysed during this study are included in this published article and its supplementary information files.
