# Supplementary material for: Chronic kidney disease mediates cardiac dysfunction associated with increased resident cardiac macrophages
Source: BMC Nephrol. 2022 Jan 28;23:47. doi: 10.1186/s12882-021-02593-7 (PMC8796634; doi:10.1186/s12882-021-02593-7)
Supplement: Supplementary file 1 — Additional file 1. [file 12882_2021_2593_MOESM1_ESM.docx]

# Supplemental Material and Methods

## Human induced pluripotent stem cell (iPSC)-derived cardiomyocytes (CM)

Human iPSC-CM were differentiated from the IMR-90 cell line1, which is derived from human foetal lung fibroblasts (Wicell, Madison, USA). Briefly, hiPSCs were plated onto 6-well plastic culture plates coated with Matrigel and maintained in E8 media (StemCell Technologies, UK). Differentiation into CM was initiated using 8 µM of CHIR99021 (Bio-Techne, UK), corresponding to Day 0 (D0). On D2, the media were replaced with RPMI plus B27 without insulin (RB-) (Thermo-Fisher Scientific, UK). On D3, cells were treated with 5 µM C59 (Bio-Techne, UK) in RB-. C59 was removed on D5, with the media replaced with RPMI without glucose. This serves as a metabolic selection for CM and ensures a high purity of CM (>95%), as non-CM are eliminated. At D15, cells were replated in culture plates and the media was replaced by RPMI plus B27 with insulin (RB+), which was then changed every other day to mature the iPSC-CMs until D30. Once matured, cells were dissociated and plated at 6.3 x 10^5^ cells/cm^2^ on 24-well culture plates coated with 0.1% gelatine. Cells were incubated for 4 days, with a media change every other day, prior to experiment.

## Human cardiac microvascular endothelial cells

Adult human cardiac microvascular endothelial cells from a healthy donor (Promocell) were cultured in EGM-MV2 medium (Promocell) with 1% penicillin/streptomycin and 10% foetal bovine serum (FBS) on 1% gelatine. Cells were seeded at 2.6 x 10^5^ cells/cm^2^ for 24 hours prior to experiment. All experiments were performed at passage 6.

## Human cardiac ventricular fibroblasts

Adult human cardiac ventricular fibroblasts were isolated from an apical biopsy collected from a patient with dilated cardiomyopathy, at Harefield Hospital (NHS, UK). Tissue samples were provided by the Cardiovascular Research Centre Biobank at the Royal Brompton and Harefield NHS Foundation Trust, UK. (NRES ethics for biobank samples: 09/H0504/104+5; Biobank approval number: NP001-06-2015 & MED_CT_17_079). Cardiac fibroblasts were isolated using the explant culture technique. Briefly, the block of tissue was trimmed of excess fat and of connective tissue, washed with PBS containing 5% penicillin/streptomycin, minced down to <10 mm3 pieces, washed again, and minced down to <1 mm3. Tissue pieces were incubated 2 minutes with 0.05% Trypsin-EDTA, which was quenched with fibroblast media (DMEM with 10% FBS and 1% penicillin/streptomycin). Tissue pieces were then placed in Petri dishes previously coated with 10 μg/ml fibronectin for 1 hour, and these were incubated for 2 hours at 37°C to allow for explant attachment to the dish. Media was then topped up until all explants were fully submerged. Media were changed after 4 days of cell outgrowth, and subsequently every other day. At confluence, fibroblasts were dissociated and replated in tissue culture flasks. Fibroblasts were used at passage 3. For experiments, fibroblasts were plated at 2.6 x 10^4^ cells/cm^2^ in 12-well tissue culture plates and cultured for 24 hours prior to experiment.

## Echocardiography in mice

Anaesthesia was induced with 4.5% isoflurane and 1.5 lit/min O2 and ophthalmic lubricant was applied (Lacrilube) to protect the corneas of anaesthetised animals from desiccation. The chests of the animals were shaved by using depilatory cream to minimize ultrasound artefacts. Anaesthetised mice were placed in a supine position on a 40°C heated platform and all four paws were fixed by using atraumatic clinical tape on the four ECG electrodes of the platform. This allowed monitoring of the mouse’s heart rate during imaging. Isoflurane dose was adjusted accordingly so that the heart rate would be maintained at 400-500 beats per minute^1,2^. Before starting imaging, warmed echo gel was applied onto the shaved chest and transducer.

Echocardiography was performed using a high-resolution VisualSonics Vevo 770 system and a 30 MHz transducer (RMV-707B). The transducer was held with the thumb of the right hand on the reference mark. The reference mark is used to orientate the image on the screen and defines the plane in which the sound leaves the transducer^3,4^.

By placing the probe on the chest of the mouse cranio-dorsally and rotated 30 º towards the right side of the neck, two-dimensional (2D) Left Ventricle (LV) long axis view was obtained^1^. This view in mice includes the LV, the aorta (Ao), the aortic valve (AV), the LV outflow tract, the Left Atrium (LA), the Mitral Valve (MV) and a slight portion of the Right Ventricle (RV)^1,5^. From this position the probe was rotated 90 º clockwise and the LV short-axis view was visualized. The resultant view includes a crescent-shaped right ventricle at the top left of the image, a circular-shaped left ventricle below the interventricular septum (IVS) and symmetrically shaped papillary muscles located in the left ventricle^1,4,5^. The shape of the left ventricle in this view is described as that of a mushroom^3^. 2D-guided LV M-mode was recorded from both long-axis view and the short-axis view at the papillary muscle level^1^.

## Echographic measurements for evaluation of systolic function

LV interventricular septal thicknesses (IVS), LV internal dimensions (LVID) and posterior wall thicknesses (PW) at diastole and systole (IVSd, LVIDd, PWd and IVSs, LVIDs, PWs, respectively) were measured from M-mode images at the level of the papillary muscles. LV ejection fraction (EF) and LV fractional shortening (FS) were measured for evaluation of LV global systolic function. LV ejection fraction (EF) and LV fractional shortening (FS) were calculated by using the following formulas^1,6–8^:

EF(%)=100x[(LVIDd^3^-LVIDs^3^)/LVIDd^3^]

FS(%)=100x[(LVIDd−LVIDs)/LVIDd]

Regional myocardial function was analysed by Doppler Tissue Imaging (DTI) obtained from parasternal short-axis views set by two-dimensional echocardiography. In mice, the only feasible and reproducible location where DTI derived parameters are measured is considered to be the posterior wall of the LV through these views^6^. The region of interest was marked to include a posterior left ventricular wall and the S' waveform of the DTI signal which represents systolic myocardial velocity was evaluated^1,5,6^. At least 3 measurements were taken for each parameter and the mean of those measurements used for grouped analysis.

1. Gao, S., Ho, D., Vatner, D. E. & Vatner, S. F. Echocardiography in Mice. in *Current Protocols in Mouse Biology* **1**, 71 (John Wiley & Sons, Inc., 2011).

2. Laakmann, S. *et al.* Minimally invasive closed-chest ultrasound-guided substance delivery into the pericardial space in mice. *Naunyn. Schmiedebergs. Arch. Pharmacol.* **386**, 227–238 (2013).

3. Boon, J. . A. *Veterinary Echocardiography*. (2011).

4. Vloumidi, E. I. & Fthenakis, G. C. Ultrasonographic examination of the heart in sheep. *Small Rumin. Res.* **152**, 119–127 (2017).

5. Respress, J. L. & Wehrens, X. H. T. Transthoracic echocardiography in mice. *J. Vis. Exp.* (2010). doi:10.3791/1738

6. Fayssoil, A. Tissue Doppler characterization of cardiac phenotype in mouse. *European Journal of Radiology* **72**, 82–84 (2009).

7. Gardin, J. M., Siri, F. M., Kitsis, R. N., Edwards, J. G. & Leinwand, L. A. Echocardiographic assessment of left ventricular mass and systolic function in mice. *Circ. Res.* **76**, 907–914 (1995).

8. Syed, F., Diwan, A. & Hahn, H. S. Murine echocardiography: A practical approach for phenotyping genetically manipulated and surgically modeled mice. *Journal of the American Society of Echocardiography* **18**, 982–990 (2005).
