# Supplementary material for: Chronic kidney disease mediates cardiac dysfunction associated with increased resident cardiac macrophages
Source: BMC Nephrol. 2022 Jan 28;23:47. doi: 10.1186/s12882-021-02593-7 (PMC8796634; doi:10.1186/s12882-021-02593-7)
Supplement: Supplementary file 2 — Additional file 2. [file 12882_2021_2593_MOESM2_ESM.docx]

# Supplemental Figures

**
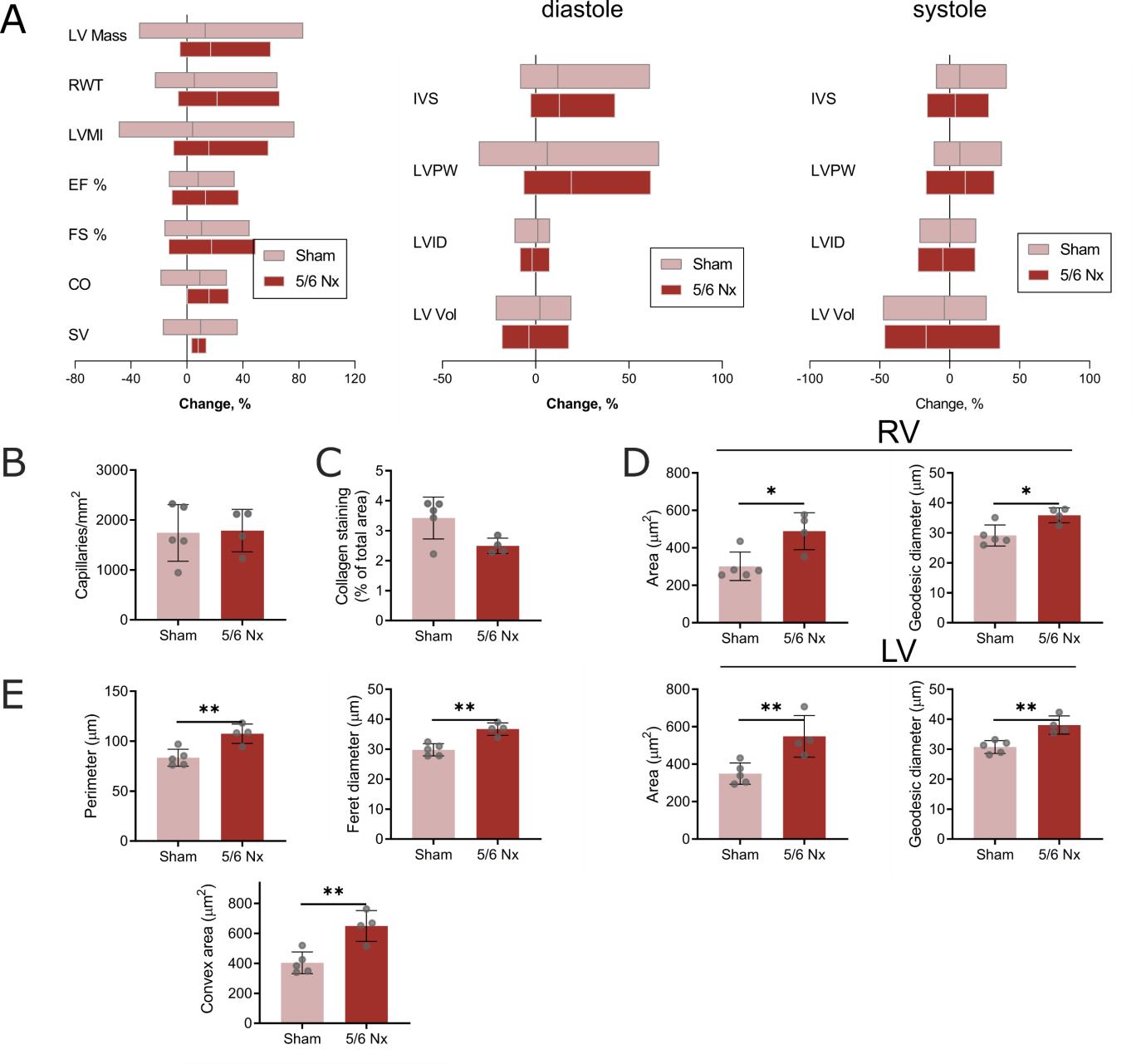
**

**Supplemental Figure 1.** A) Mice underwent sham or 5/6 nephrectomy (Nx) and echocardiography performed at 6 and 12 weeks (see methods) and percentage of change was calculated. Left panel shows changes in left ventricular (LV) mass, relative wall thickness (RWT), left ventricular mass index (LVMI), ejection fraction (EF), fractional shortening (FS), cardiac output (CO) and stroke volume (SV). Middle and right panels show diastolic/systolic left ventricular posterior wall (LVPW) thickness, intraventricular septum (IVS) thickness, left ventricular internal diameter (LVID) and left ventricular volume (LV Vol). B) Number of blood vessels per mm^2^ of heart tissue quantified in sham and 5/6 Nx mice. C) Collagen content measured by visualising PSR under polarised light in sham and 5/6 Nx mice. D) Left ventricle (LV) and right ventricle (RV) cardiomyocyte cell areas and geodesic diameters (length of the shortest path between two furthest points within a region) in sham and 5/6 Nx mice measured on A488-WGA stained heart slices. Each point represents the mean of at least 300 to 3000 cardiomyocytes per animal (two-tailed unpaired t-test, * P<0.05, ** P<0.01).E) Total heart cardiomyocyte perimeter, ferret diameter (maximum length) and convex area measured in sham and 5/6 Nx mice measured on A488-WGA stained heart slices. Each point represents the mean of at least 300 to 3000 cardiomyocytes per animal (two-tailed unpaired t-test, ** P<0.01).

**
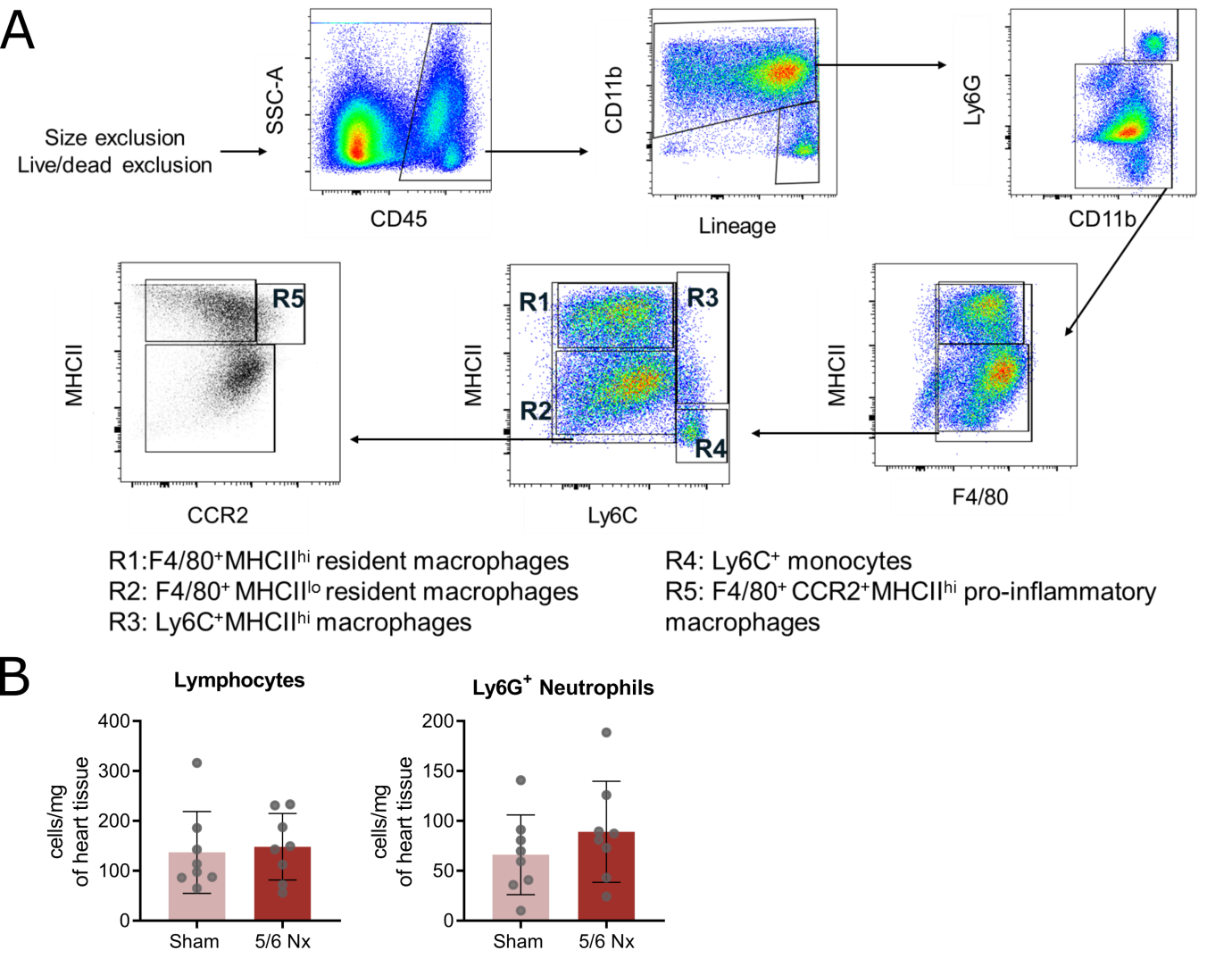
**

**Supplemental Figure 2.** A) Gating strategy for heart cells in flow cytometry. Briefly, after gating on CD45^+^ leukocytes, lymphocytes (lineage) and neutrophils (Ly6G) were excluded from myeloid cells (CD11b). Macrophage and monocyte subpopulations were analysed using F4/80, MHCII, Ly6C and CCR2 staining. B) Lymphocytes and neutrophils numbers per mg of heart tissue and proportion (stacked histograms) in sham and 5/6 nephrectomised (Nx) mice.


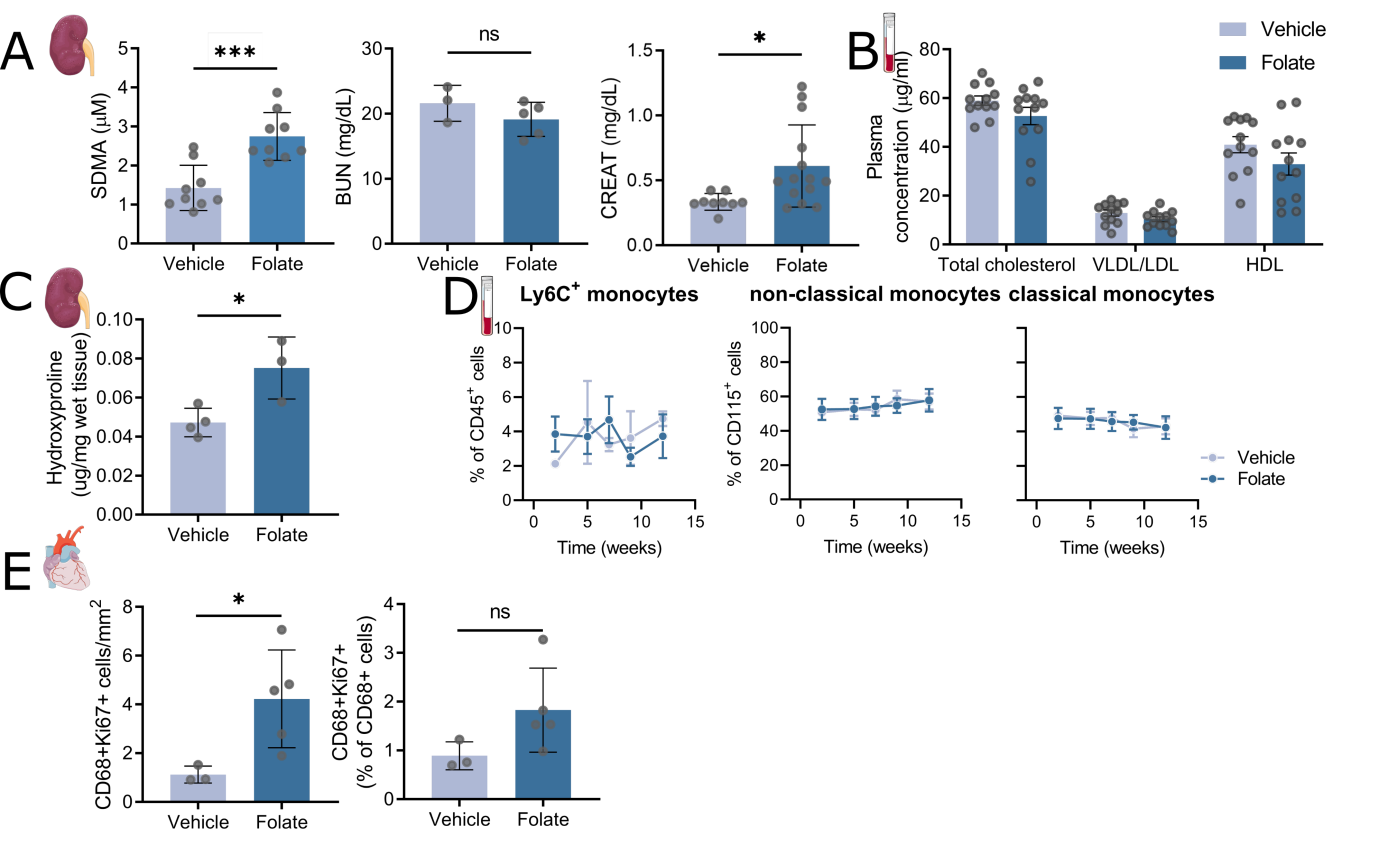


**Supplemental Figure 3.** A) Plasma SDMA, BUN and creatinine (CREAT) levels in levels in vehicle control and folate treated mice after 12 weeks. Each point represents individual mice (two-tailed unpaired t-test, * P<0.05, *** P<0.001). B) Plasma cholesterol levels in vehicle control and folate treated mice after 12 weeks. Presented as total cholesterol (TC), very low/low-density lipoproteins (VLDL/LDL) and high-density lipoproteins (HDL). Each point represents individual mice either injected with vehicle or folate. C) Kidney tissue homogenates were collected from 12 weeks vehicle control or folate treated animals and fibrosis quantitatively analysed using 4-hydroxyproline colorimetric assay (two-tailed unpaired t-test, * P<0.05). D) Total blood monocyte and monocyte subset frequency over 12 weeks in vehicle control or folate treated animals. Presented as percentage of all leukocytes (total monocytes) or of all CD115+ monocytes for classical monocytes or non-classical monocytes. Mean±SD at each time point. n=12. E) Proportion and number per mm^2^ of Ki67-positve macrophages in CD68-positive macrophages measured in whole heart slice in vehicle or folate-treated animals (two-tailed unpaired t-test, * P<0.05).


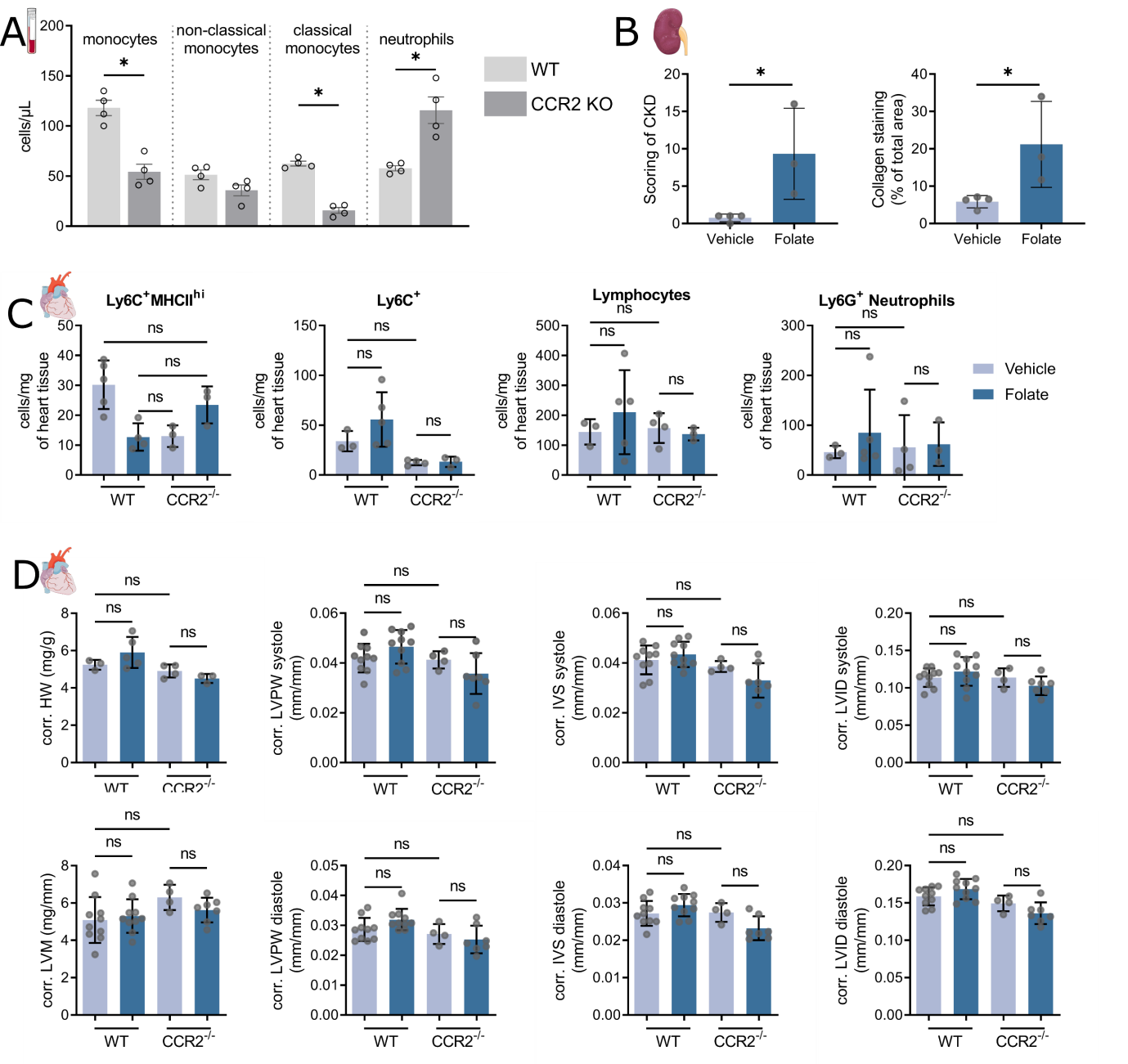


**Supplemental Figure 4.** A) Number of cells (monocytes, classical and non-classical monocytes, neutrophils) per µL of blood in CCR2^+/+^ (WT) and CCR2^-/-^ mice at baseline (* P<0.05). B) Kidney collagen content as measured by colour deconvolution and CKD score (see materials and methods) in vehicle and folate treated CCR2^-/-^ mice (two-tailed unpaired t-test, * P<0.05). C) Cardiac F4/80^+^MHCII^hi^ and F4/80^+^MHCII^lo^ resident macrophage, Ly6C^+^MHCII^hi^ macrophage, or Ly6C^+^ monocyte numbers per mg of heart tissue in vehicle control or folate treated WT and CCR2^-/-^ mice (Kruskal-Wallis test and Dunn’s post-hoc). D) Cardiac remodelling measured by in vehicle control or folate treated WT and CCR2^-/-^ mice showing heart weight (HW), left ventricular mass (LVM), diastolic/systolic left ventricular posterior wall (LVPW) thickness, intraventricular septum (IVS) thickness, and left ventricular internal diameter (LVID). Parameters are normalised to tibia length, expect heart weight to body weight (Kruskal-Wallis test and Dunn’s post-hoc).


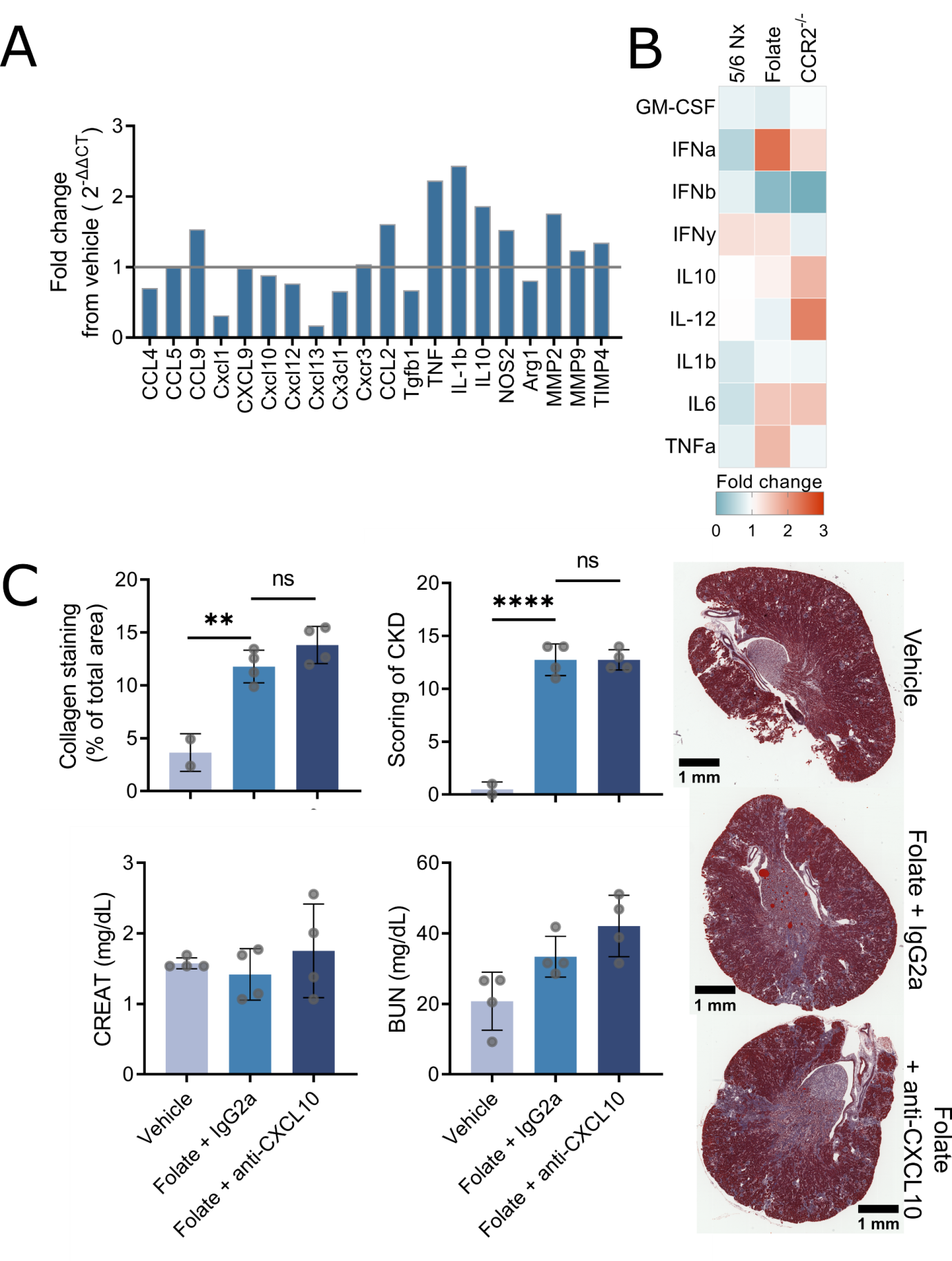


**Supplemental Figure 5.** A) A pool of macrophages was isolated from hearts from vehicle control or folate treated (12 weeks) mice. Data shown is extracellular matrix, inflammatory and chemokine gene expression presented as fold change from control. B) Heatmap showing fold change in plasma levels of cytokines in 5/6 nephrectomised mice, folate treated wildtype and folate treated CCR2^-/-^ mice (to their respective controls). C) Top panels show kidney collagen content as measured by colour deconvolution along with representative images of kidney stain with Masson’s Trichrome and CKD score (see materials and methods) in vehicle injected with PBS and folate treated injected with anti-CXCL10 antibody or control IgG2a. Bottom panels show plasma BUN and creatinine (CREAT) levels at 12 weeks (Kruskal-Wallis test and Dunn’s post-hoc, ** P<0.01, **** P<0.0001).


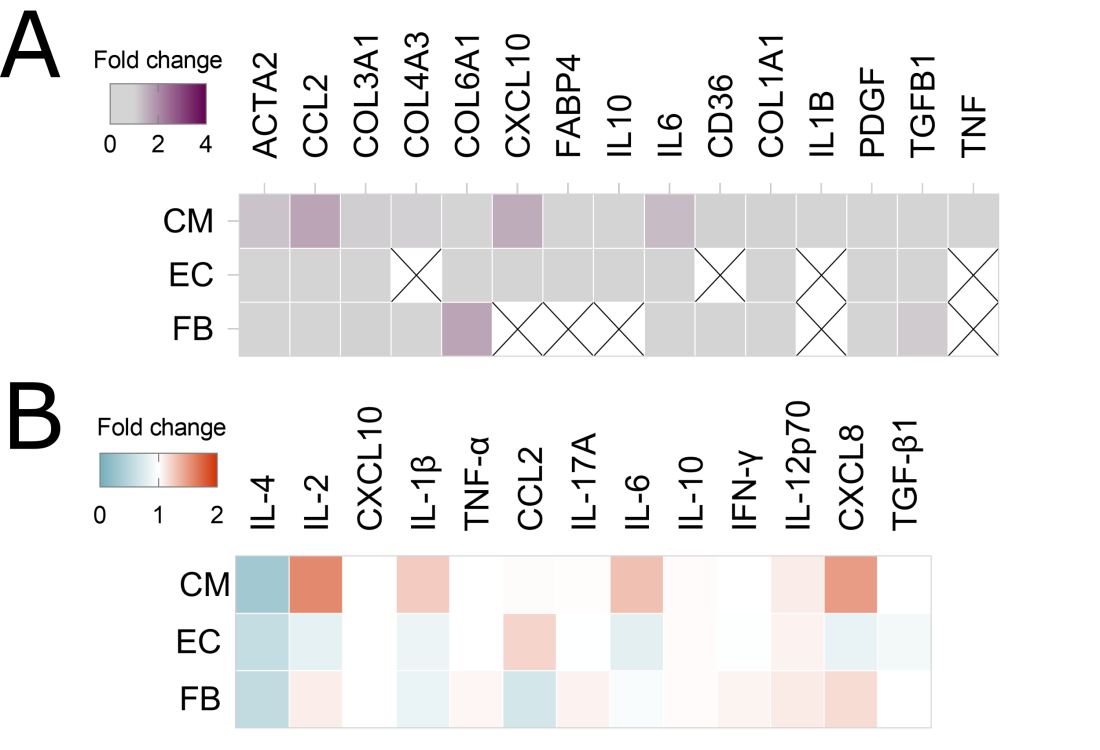


**Supplemental Figure 6.** A) Heatmap showing mRNA expression fold change from control in iPSC-derived cardiomyocytes (CM), human cardiac microvascular endothelial cells (EC) and primary ventricular fibroblasts (FB) treated with serum from healthy controls. C) Heatmap showing protein secretion fold change from control in supernatant of iPSC-CM, EC and FB treated with serum from healthy controls.


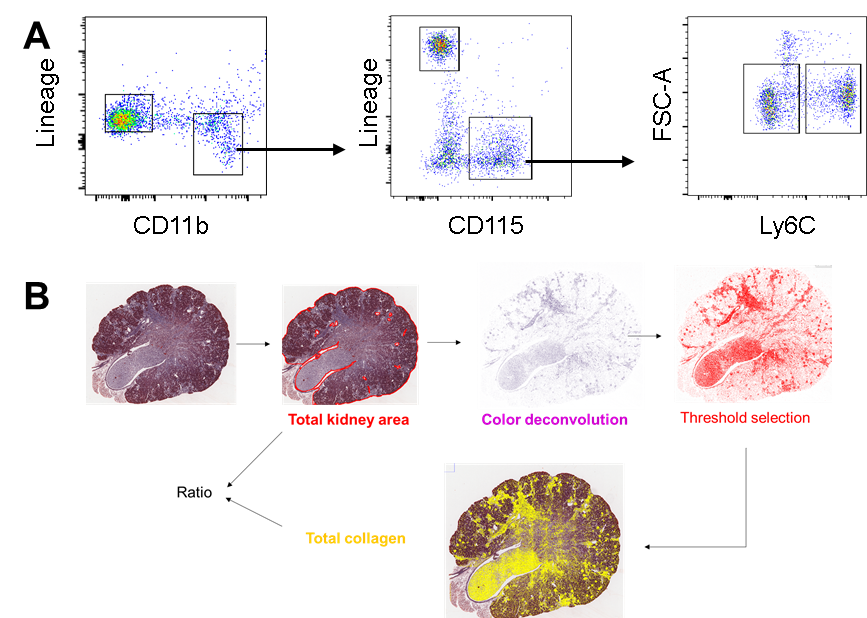


**Supplemental Figure 7.** A) Gating strategy for blood monocytes. B) Colour deconvolution for total collagen kidney analysis.

**Supplemental Table 1.** Details of antibodies used in flow cytometry, cell sorting and immunohistochemistry.

| **Target** | **Clone** | **Supplier** |
| --- | --- | --- |
| **Biot. CD3e** | 145-2C11 | BD Biosciences |
| **Biot. CD19** | 1D3 | BD Biosciences |
| **Biot. NK1.1** | PK136 | BD Biosciences |
| **F4/80** | A3-1 | Biorad |
| **F4/80** | BM8 | BioLegend |
| **Ly6G** | 1A8 | BioLegend |
| **CD45** | 30-F11 | BioLegend |
| **Ly6C** | HK1.4 | BioLegend |
| **CD11b** | M1/70 | BD Biosciences |
| **MHCII** | AF6-120.1 | eBioscience |
| **CCR2** | 475301 | R&D |
| **CD115** | AFS98 | BioLegend |
| **CD11c** | N418 | BioLegend |
| **CD68** | FA-11 | BioLegend |
| **Ki67** | SP6 | Abcam |

**Supplemental Table 2.** Details of primers used for mouse qPCR.

| **mRNA** | **Accession Nr** | **Forward** | **Reverse** |
| --- | --- | --- | --- |
| **ACTA1** | NM_001272041 | CGCCAGCCTCTGAAACTAGA | ACGATGGATGGGAACACAGC |
| **ACTA2** | NM_007392 | GCCATCTTTCATTGGGATGGA | CCCCTGACAGGACGTTGTTA |
| **ACTC1** | NM_009608 | CCCCGTCCATCAGAGAGCTA | GGTTCTGTAGGCGTGCTAGG |
| **AGT** | NM_007428 | GTTGGCGCTGAAGGATACACA | GACCCAGGTCAAGATGCAGAA |
| **ARG1** | NM_007482 | GTACATTGGCTTGCGAGACG | TTTTCTTCCTTCCCAGCAGGT |
| **CAMK2D** | NM_001025438 | GCTCTTTGAGGAGCTCGGAA | TGATGGTCCCTAGCAGAAAGC |
| **CCL19** | NM_011888 | GGGGTGCTAATGATGCGGAA | GTGGTGAACACAACAGCAGG |
| **CCL2** | NM_011333 | CACTCACCTGCTGCTACTCA | GCTTGGTGACAAAAACTACAGC |
| **CCL20** | NM_016960 | CACAAGACAGATGGCCGATG | CAGCCCTTTTCACCCAGTTC |
| **CCL4** | NM_013652 | CCCAGCTCTGTGCAAACCTA | CCATTGGTGCTGAGAACCCT |
| **CCL5** | NM_013653 | CTGCTGCTTTGCCTACCTCT | CGAGTGACAAACACGACTGC |
| **CCL9** | NM_011338 | GCCCAGATCACACATGCAAC | AGGACAGGCAGCAATCTGAA |
| **CD163** | [NM_001170395](http://www.ncbi.nlm.nih.gov/nuccore/NM_001170395) | AGGTGCTGGATCTCCTGGTTG | AGGAGCGTTAGTGACAGCAG |
| **CD31** | several | CACACCGAGAGCTACGTCAT | TTGGATACGCCATGCACCTT |
| **CD36** | NM_001159555.1 | GGAGCAACTGGTGGATGGTT | CTACGTGGCCCGGTTCTAAT |
| **CD68** | NM_001291058 | GGGGCTCTTGGGAACTACAC | ATGCCCCAAGCCTTTCTTCC |
| **COL1A2** | NM_007743 | AGGAAAGAGAGGGTCTCCCG | GCCAGGAGGACCCATTACAC |
| **COL3A1** | NM_009930 | TGACTGTCCCACGTAAGCAC | GAGGGCCATAGCTGAACTGA |
| **CX3CL1** | NM_009142 | GCGACAAGATGACCTCACGA | TGTCGTCTCCAGGACAATGG |
| **CXCL1** | NM_008176 | CGCCTATCGCCAATGAGCTG | GAGTGTGGCTATGACTTCGGT |
| **CXCL10** | NM_021274 | TCTGAGTGGGACTCAAGGGAT | AGGCTCGCAGGGATGATTTC |
| **CXCL11** | NM_019494.1 | CAGCTGCTCAAGGCTTCCTTA | CAACTTTGTCGCAGCCGTTA |
| **CXCL12** | NM_021704.3 | GCTCTGCATCAGTGACGGTA | TAATTTCGGGTCAATGCACA |
| **CXCL13** | NM_018866 | CTCCAGGCCACGGTATTCTG | CCAGGGGGCGTAACTTGAAT |
| **CXCL4** | NM_019932.4 | AGCTCATAGCCACCCTGAAGA | TGACATTTAGGCAGCTGATACC |
| **CXCL9** | NM_008599.4 | TCGGACTTCACTCCAACACAG | AGGGTTCCTCGAACTCCACA |
| **CXCR3** | NM_009910.3 | GCCATGTACCTTGAGGTTAGT | GGGAGTCAGAGAAGTCGCTC |
| **F4/80** | NM_010130 | CCATCCACTTCCAAGATGGGTTA | TGCCATCAACTCATGATACCCT |
| **HPRT** | NM_013556 | CAGTCCCAGCGTCGTGATTA | TGGCCTCCCATCTCCTTCAT |
| **IL-10** | NM_010548 | CAGCCGGGAAGACAATAACTG | CCGCAGCTCTAGGAGCATG |
| **IL17RA** | NM_008359.2 | CCCAAGCCAGTTGCAGACTAC | CAGAAAGCCTCCAGGTCATACA |
| **IL-1B** | NM_008361 | CAACCAACAAGTGATATTCTCC | GATCCACACTCTCCAGCTGCA |
| **INOS** | NM_010927 | GAAACTTCTCAGCCACCTTGG | TCCAACGTTCTCCGTTCTCTTG |
| **MMP-2** | NM_008610 | CCTGTTCAACGGTCGGGAAT | GGTAAACAAGGCTTCATGGGG |
| **MMP-9** | NM_NM_013599.4 | CTCTCCTGGCTTTCGGCTG | AGCGGTACAAGTATGCCTCTG |
| **NPPA** | NM_008725 | TTTCAAGAACCTGCTAGACCACC | CTCGGGGAGGGAGCTAAGT |
| **NPPB** | NM_008726 | TTTGGGCTGTAACGCACTGA | CACTTCAAAGGTGGTCCCAGA |
| **TGFB1** | NM_011577 | ACTGGAGTTGTACGGCAGTG | GGCTGATCCCGTTGATTTCC |
| **TIMP-1** | NM_001044384.1 | GGCATCTGGCATCCTCTTGT | TAGCCCTTATGACCAGGTCCG |
| **TIMP4** | NM_080639 | TGTGCAACTACATTGAGCCCT | GCAAGTGGTGATTTGGCAGC |
| **TNF** | NM_013693 | CATCTTCTCAAAATTCGAGTGACAA | TGGGAGTAGACAAGGTACAACCC |
| **VIM** | NM_011701.4 | GGCTCGTCACCTTCGTGAAT | AGGCAGAGAAATCCTGCTCTC |

**Supplemental Table 3.** Details of primers used for human qPCR.

| **mRNA** | **Accession Nr** | **Forward** | **Reverse** |
| --- | --- | --- | --- |
| **ACTA2** | NM_001613 | GGTGCTGTCTCTCTATGCCT | GACAATCTCACGCTCAGCAG |
| **CD36** | NM_001001547 |  |  |
| **COL1A1** | NM_000088.3 | GTCTTTTGCTTCCTCCCACC | TCCGACCTCTCTCCTCTAA |
| **COL3A1** | NM_000090 | CGGCAATCCTGAACTTCCTG | ATCAGCTTCAGGTCCTTCTT |
| **COL4A3** | NM_000091 | GGGACCACTCCAGACCTCA | TTAAAAAGAGGCGGATGG |
| **COL6A1** | NM_001848 | GGCTTCATCGACAACCTGAG | TCCAGCCCCTTCTTGATAGC |
| **CXCL10** | NM_001565 | GGCATTCAAGGAGTACCTCT | TCATTGTAGCAATGATCTC |
| **CCL2** | NM_002982 | TCAAACTGAAGCTCGCACTCT | GGGGCATTGATTGCATCTGG |
| **FABP4** | NM_001442 | TTCTGCACATGTACCAGGACAC | GCCAGGAATTTGACGAAGTCAC |
| **GAPDH** | NM_001357943.2 | TGCACCACCAACTGCTTAGC | GCATGGACTGTGGTCATGAG |
| **IL-10** | NM_000572.3 | GCCTAACATGCTTCGAGATC | TGATGTCTGGGTCTTGGTTC |
| **IL-6** | NM_001371096.1 | CCTCAAACTCCAAAAGACCAGTGATG | TGTGTGAAAGCAGCAAAGAGGC |
| **IL-1B** | NM_000576.3 | GAAGCTGATGGCCCTAAACA | AAGCCCTTGCTGCTGTAGTGGTG |
| **PDGF** | NM_002607 |  |  |
| **TGFB1** | NM_000660.7 | AGTGAACCCGTTGATGTCCA | GATGTACCGGAGTTGTGCG |
| **TNF** | NM_000594.4 | GCCAGAGGGCTGATTAGAGA | TCAGCCTCTTCTCCTTCCTG |

**Supplemental Table 4.** Details of 37 CKD patients recruited to study.
